# Phagocytosis of necroptotic cells optimizes type 1 conventional dendritic cells for induction of a cytotoxic T-cell response

**DOI:** 10.1038/s41418-026-01689-7

**Published:** 2026-02-28

**Authors:** Mo D. Staal, Zhixian Wang, Douwe M. T. Bosma, Julia Busselaar, Ellen Schrama, Evert de Vries, Yanling Xiao, Jannie Borst

**Affiliations:** 1 Department of Immunology, Leiden University Medical Center, Leiden, The Netherlands; 2 Oncode Institute, Leiden University Medical Center, Leiden, The Netherlands

**Keywords:** Cell death and immune response, Antigen-presenting cells

## Abstract

Whether a T-cell response to dead cells arises depends on whether they harbor recognizable antigens, but also how dead cell debris impacts conventional dendritic cells (cDCs). In all tissues, cell debris is continuously phagocytosed by cDCs that can migrate to lymph nodes (LNs) and initiate T-cell responses, depending on their maturation state. The cDC1 lineage excels at antigen cross-presentation, which is required for induction of the CD8^+^ cytotoxic T-lymphocyte (CTL) response. At steady state, cDC1s undergo homeostatic maturation, which leads to T-cell non-responsiveness. This cDC1 state has recently been defined by ex vivo transcriptomics as resulting from phagocytosis of apoptotic cell debris. Necroptotic (tumor) cell death has been described as more immunogenic than apoptotic cell death, but its impact on cDC maturation has not been defined. In this study, we use an in vitro model to compare side-by-side the impact of well-defined apoptotic versus necroptotic tumor cell debris on the CTL response induced by cDCs, as well as on phenotype and function of both cDC1s and cDC2s. We confirm that cDC2s are less efficient than cDC1s in dead cell phagocytosis and find that they minimally respond to it in terms of gene expression. Apoptotic cell debris is more efficiently phagocytosed by cDC1s than necroptotic cell debris and induces a cDC1 cell state in vitro that has an evident transcriptomic relationship to the homeostatic maturation state defined ex vivo, thus validating our approach. We identify necroptosis as more potent than apoptosis in inducing a CTL response and attribute this to the ability of necroptotic cell debris to induce a specific, transcriptomically defined maturation state in cDC1s, characterized by cytokine and phosphoinositide signaling, cytoskeletal and metabolic activity and functional differentiation. We suggest that this cDC1 signature may be used to diagnose immunogenicity of necroptosis ex vivo.

## Introduction

The idea to modulate regulated cell death (RCD) to direct immunity in cancer or other diseases is attractive, but rational intervention requires further understanding of how different cell death modalities impact immune regulation [1]. In general terms, cell death may be immunologically silent, proinflammatory, or immunogenic, wherein immunogenicity is defined as the ability to invoke a T-cell response [2]. The process of T-cell priming relies on cDCs that drive CD8^+^ and CD4^+^ T-cell proliferation and effector differentiation by presenting antigens and providing essential costimulatory and cytokine signals [3–5]. At steady-state, as well as during infection or other immunological challenges, cDCs phagocytose cell debris in peripheral tissues and traffic to draining lymph nodes (LNs) [6], guided by the CCR7 chemokine receptor [4]. There, cDCs present peptides generated from ingested proteins on major histocompatibility (MHC) molecules to naïve T cells. Among the two cDC lineages, cDC1s are best equipped to phagocytose particulate cell debris, process it into peptides (antigens) via the cross-presentation pathway and present these in MHC-I to CD8^+^ T cells [7]. The cDC2 lineage is hallmarked by expression of SIRPα [8], the receptor for the “don’t eat me” signal CD47 [9, 10]. Therefore, cDC2s are more specialized in processing soluble molecules in the endolysosomal system and presenting these in MHC-II to CD4^+^ T cells.

Cell death will lead to release of antigens that may be recognizable by T cells, but whether a T-cell response ensues depends on the ability of dead cells to invoke an immunogenic cDC state [4, 11]. Under homeostatic conditions, apoptotic cell death ensures the immunologically silent removal of damaged and unwanted cells during remodeling and turnover of healthy tissues. Apoptotic cells are phagocytosed by cDC1s that subsequently undergo homeostatic maturation, allowing them to express CCR7 and migrate to LNs [6, 12, 13]. Under these conditions, cDCs maintain tolerance to self-antigens by preventing activation of sporadic self-reactive T cells, both directly and via regulatory T-cell (Treg) activity [11, 14]. During infection or cancer, other modes of RCD may come into play, specifically necroptosis and pyroptosis. These are lytic forms of cell death that are considered to be more inflammatory, due to the release of intracellular content, as well as the production of pro-inflammatory IL-1β and IL-18 in the case of pyroptosis [15]. Interestingly, these modes of cell death have been acquired later in evolution and do not play a role in normal tissue homeostasis, but rather in the defense against infection [15].

It is generally accepted in the field that stimulation of cDCs by specific pathogen-associated molecular patterns (PAMPs) converts them from a tolerogenic to an immunogenic state [12, 13]. cDCs express a multitude of pattern recognition receptors (PRRs), including Toll-like receptors (TLRs), that via well-defined intracellular signaling pathways lead to cDC maturation. The (potential) immunogenicity of cell death under non-infectious conditions such as cancer has been associated with the release of damage-associated molecular patterns (DAMPs) [2]. DAMPs are emitted by stressed and dying cells and can likewise bind to a variety of PRRs, including TLRs, cGAS, ZBP-1, as well as RIG-l and NOD-like receptors [16]. Several DAMPs were shown to contribute to immunogenicity of cancer cells after induction of cancer cell death by specific drugs and ionizing radiation [2, 17, 18]. Proteomic analysis has shown that apoptosis or necroptosis of the same cell type can be discerned based on release of specific intracellular contents [19], but classically defined DAMPs are not key discriminating factors [20]. DAMPs and other soluble factors released by dying cells may act as “find me” and “eat me” signals, promoting phagocytosis, or directly promote the immunogenic state of antigen presenting cells [2]. Thus far, however, it is unclear which signals define the immunogenic state of cDCs after different forms of RCD.

It is therefore important to study the impact of dead/dying cells on cDCs. Transcriptional profiling has pointed out that cDC1s matured under either homeostatic or PRR-induced immunogenic conditions share a set of maturation genes and both upregulate CCR7 and costimulatory molecules CD80, CD86, and CD40 [12, 13]. Under homeostatic conditions, however, regulatory T cells (Tregs) attenuate the costimulatory capacity of cDCs [21], while under immunogenic conditions this activity is overruled. Discerning features between tolerogenic and immunogenic cDC1s are expression of a cholesterol biosynthesis program versus a type I IFN signature [12, 13], but how this difference impacts Treg activity is not known. Thus, it is of great interest to further define the effect of different RCD modalities on cDC maturation states, in order to understand the immunogenic switch.

We here compared the impact of apoptotic versus necroptotic tumor cells on murine cDC1s and cDC2s and the ability of cDCs to induce a CTL response. Necroptosis, as triggered by e.g. death receptors or PAMPs, impinges on pore formation in cellular membranes by mixed lineage kinase domain-like pseudokinase (MLKL), which lies downstream of receptor-interacting serine/threonine-protein kinase (RIPK)3 [22], the central mediator of necroptosis [23]. Previous studies in mice have shown that necroptotic tumor cells were more immunogenic than apoptotic tumor cells, as read out by induction of a CD8^+^ T-cell response, tumor elimination and generation of immunological memory [24–28]. The T-cell response relied on cDC1s, but impact on cDC phenotype and gene expression was not determined. Therefore, we examined side-by-side how tumor cells dying via apoptosis or necroptosis affect state and function of murine cDCs, using cellular assays, flow cytometry and transcriptomics. Our findings highlight the unique ability of necroptotic cells to induce a specific maturation state in cDC1s rather than cDC2s, which is associated with superiority in CTL priming.

## Results

### Validation of ligand-free inducible models for apoptotic or necroptotic tumor cell death

**Fig. 1:**
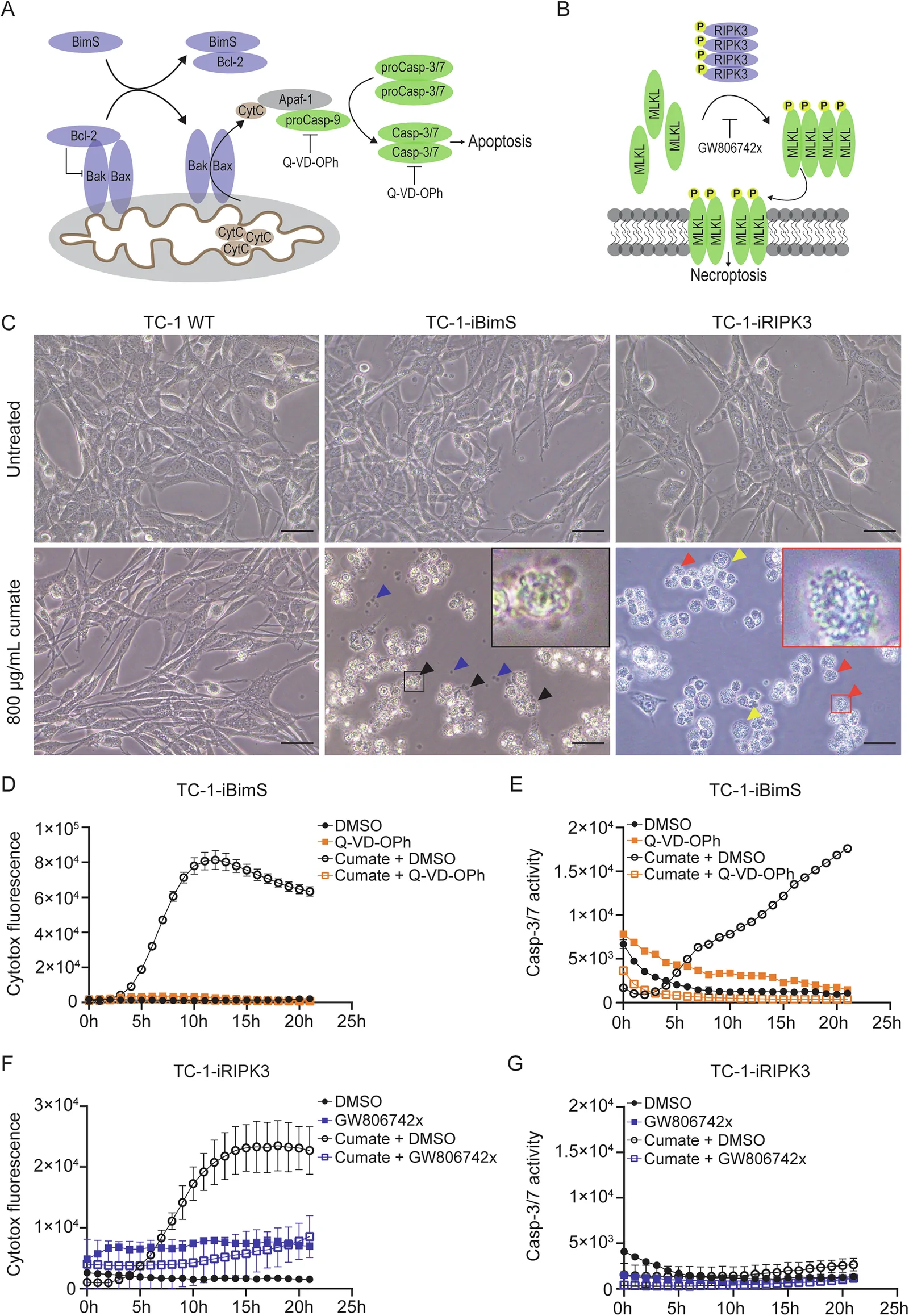
Validation of inducible apoptosis or necroptosis in engineered TC-1 tumor cells. **A, B** Graphical illustrations of signaling pathways involved upon cumate-induced overexpression of BimS leading to apoptosis **A**, or RIPK3 leading to necroptosis **B**. **C** Representative light microscopy images of indicated TC-1 cells, untreated or treated with cumate for 24 h. Blue arrowheads: apoptotic bodies; black arrowheads and magnified black inlay: membrane blebbing; yellow arrowheads: cell swelling; red arrowheads and magnified red inlay: dying cell morphology. Scale bar represents 40 µm. **D–G** Real-time Incucyte analysis of Cytotox Dye incorporation **D, F** and Caspase-3/7 activity **E, G** in TC-1-iBimS and TC-1-iRIPK3 cells during indicated treatments. Means ± SD of technical triplicates are shown, representative of two independent experiments.

As a model system, we used the murine lung carcinoma cell line TC-1 [29] that we genetically engineered to conditionally overexpress either BH3-only protein BimS (TC-1-iBimS) to induce apoptosis [30] (Fig. 1A), or a homo-oligomerizing form of RIPK3 (TC-1-iRIPK3) to induce necroptosis (Fig. 1B), by use of the cumate gene switch (Fig. S1A) [31]. After overnight gene induction with cumate, morphology-based analysis of dying TC-1-iBimS cells revealed clear characteristics of apoptosis, such as membrane blebbing and apoptotic bodies (Fig. 1C). BimS overexpression led to increased plasma membrane permeability as measured by Cytotox Dye incorporation (Fig. 1D, Movie 1), Caspase-3/7 activity as measured with fluorogenic substrate (Fig. 1E) and phosphatidylserine exposure as measured by Annexin V fluorescent dye (Fig. S1B), which were fully blocked by pan-caspase inhibitor Q-VD-OPh (Fig. 1D, Movie 2, Fig. 1E, Fig. S1B). Dying TC-1-iRIPK3 cells became swollen and remained mostly as one entity without clear cellular structures (Fig. 1C), indicating necroptotic cell death. Furthermore, RIPK3 overexpression resulted in loss of membrane integrity as shown by Cytotox Dye incorporation (Fig. 1F, Movie 3) and phosphatidylserine exposure, which were blocked by MLKL inhibitor GW806742x (Fig. 1F, Fig. S1C, Movie 4). Confirming the necroptotic nature of RIPK3-induced cell death, Caspase-3/7 activity was absent (Fig. 1G) and pan-caspase inhibition with Q-VD-OPh did not affect dying TC-1-iRIPK3 cells (Fig. S1D). Cell death of TC-1-iBimS cells was delayed by GW806742x, but not inhibited (Fig. S1E, Movie 5), excluding MLKL involvement. Taken together, we generated and validated TC-1 tumor cell lines to conditionally undergo apoptosis or necroptosis upon cumate-induced expression of either BimS or oligomerizing RIPK3.

### Uptake of necroptotic cells endows cDCs with optimal ability to induce CD8^+^ T-cell expansion

**Fig. 2:**
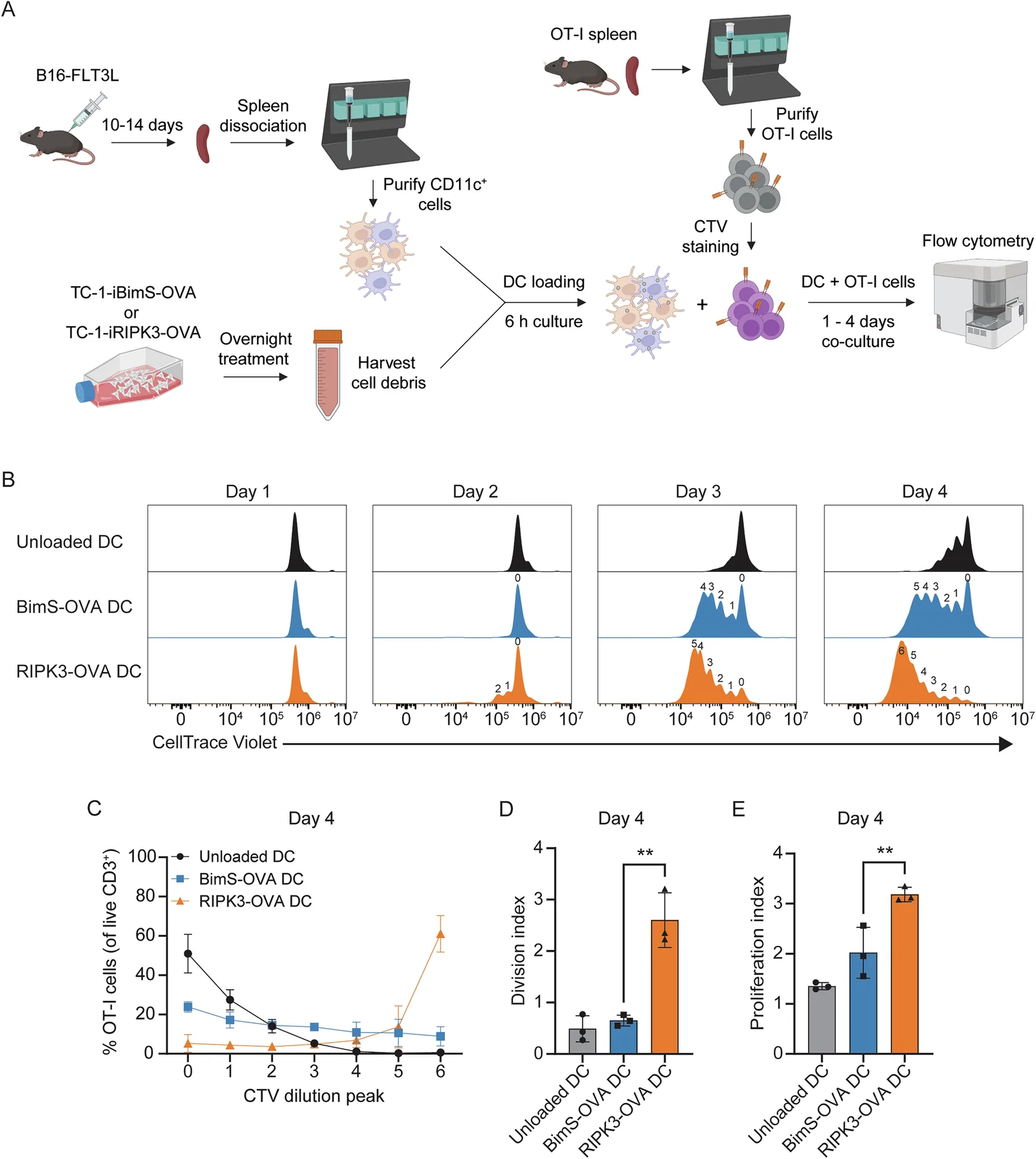
OVA-specific CD8^+^ T-cell proliferation induced by cDCs after phagocytosis of apoptotic or necroptotic TC-1-OVA cells. **A** Experimental design of the cDC-OT-I T cell co-culture platform. Created in BioRender. OVA-expressing TC-1 cells were treated overnight with cumate and cell debris (medium) was added at a 1:1 ratio of plated TC-1 cells to cDCs. As control, cDCs were loaded with 250 μg/ml sOVA. OT-I T cells and loaded cDCs were co-cultured at a 25:1 ratio. **B** Representative flow cytometry histograms showing CellTrace Violet (CTV) dilution in OT-I cells after 1, 2, 3, and 4 days of co-culture with indicated cDCs. Numbers in the histograms indicate CTV dilution peaks. **C** Frequency of OT-I cells in each CTV dilution peak after 4 days of co-culture with indicated cDCs. Division index **D** and proliferation index **E** of the OT-I cell population after 4 days of co-culture with indicated cDCs. Means ± SD of two independent experiments are shown in **C** and means ± SD of three independent experiments in **D** and **E**. **P < 0.01; one-way ANOVA with Tukey’s multiple comparisons test.

We next investigated the impact of apoptotic versus necroptotic cell death on T-cell priming in an in vitro assay, using primary cDCs and T cells from mice. Since cDCs occur in very low frequencies, we s.c. implanted in mice B16 tumor cells expressing FLT3 ligand, which stimulates cDC generation from progenitor cells [32]. The cDCs were enriched by magnetic sorting from spleens based on CD11c expression [33] and used with a purity of 85% or higher, with an average cDC1 to cDC2 ratio of 1:1 (Fig. S2). With these cDCs, we first set up and validated a priming platform with full-length chicken ovalbumin (OVA) as model antigen. Choice of this antigen enabled use of OT-I responder CD8^+^ T cells that express a transgenic TCR recognizing OVA_257-264_ peptide in the context of MHC-I (H-2K^b^) (Fig. 2A). Purified primary cDCs were loaded for 6 h ex vivo with soluble (s) full-length OVA, after which CellTrace Violet (CTV)-labeled OT-I cells were added, and cells were co-cultured for 1-4 days. The cDCs loaded with sOVA could stimulate OT-I proliferation, as shown by CTV dilution (Fig. S3A). This response depended on cDCs, as OT-I cells incubated with sOVA in absence of cDCs did not proliferate (Fig. S3B). We next introduced a cDNA construct encoding full-length OVA in TC-1-iBimS and TC-1-iRIPK3 cells (Fig. S3C), to enable read out of the OT-I T cell response to dying tumor cells.

After this validation, ex vivo cDCs were loaded for 6 h with either apoptotic or necroptotic tumor cells and co-cultured with CTV-labeled OT-I cells (Fig. 2A). For this purpose, the medium overlaying the dead/dying TC-1 cells was harvested by pipetting and added as such, without centrifugation, in equal volumes to the cDCs. Both BimS-OVA DCs and RIPK3-OVA DCs induced OT-I proliferation, as shown by CTV dilution over time (Fig. 2B). This response was fully dependent on cDCs (Fig. S3B). More OT-I cells entered the cell cycle and proliferation was more advanced when induced by RIPK3-OVA DCs compared to BimS-OVA DCs (Fig. 2B). This was likely not due to higher antigenic load, since OVA protein levels were lower in TC-1-iRIPK3-OVA cells than in TC-1-iBimS-OVA cells (Fig. S3C) and total protein amount in medium from TC-1-iBimS-OVA cells was lower when compared to TC-1-iRIPK3-OVA cells (Fig. S3D). At day 4 after stimulation with RIPK3-OVA DCs, almost all OT-I cells had entered the cell cycle and the largest proportion had already reached maximum CTV dilution (Fig. 2B, C). In contrast, after stimulation with BimS-OVA DCs, more than 20% of OT-I cells had not entered the cell cycle, and only a small proportion had reached maximum CTV dilution (Fig. 2B, C). The division index of OT-I cells, calculated as the average number of divisions of each cell in the original population, was significantly higher after stimulation by RIPK3-OVA DCs than by BimS-OVA DCs (Fig. 2D). Likewise, the proliferation index was higher, calculated as the average number of divisions undergone by cells that had entered cell cycle (Fig. 2E). These results show that cDCs that have phagocytosed necroptotic cells are more proficient in inducing clonal expansion of antigen-specific CD8^+^ T-cells than cDCs that have phagocytosed apoptotic tumor cells.

### Uptake of necroptotic cells endows cDCs with optimal ability to induce CTL differentiation

**Fig. 3:**
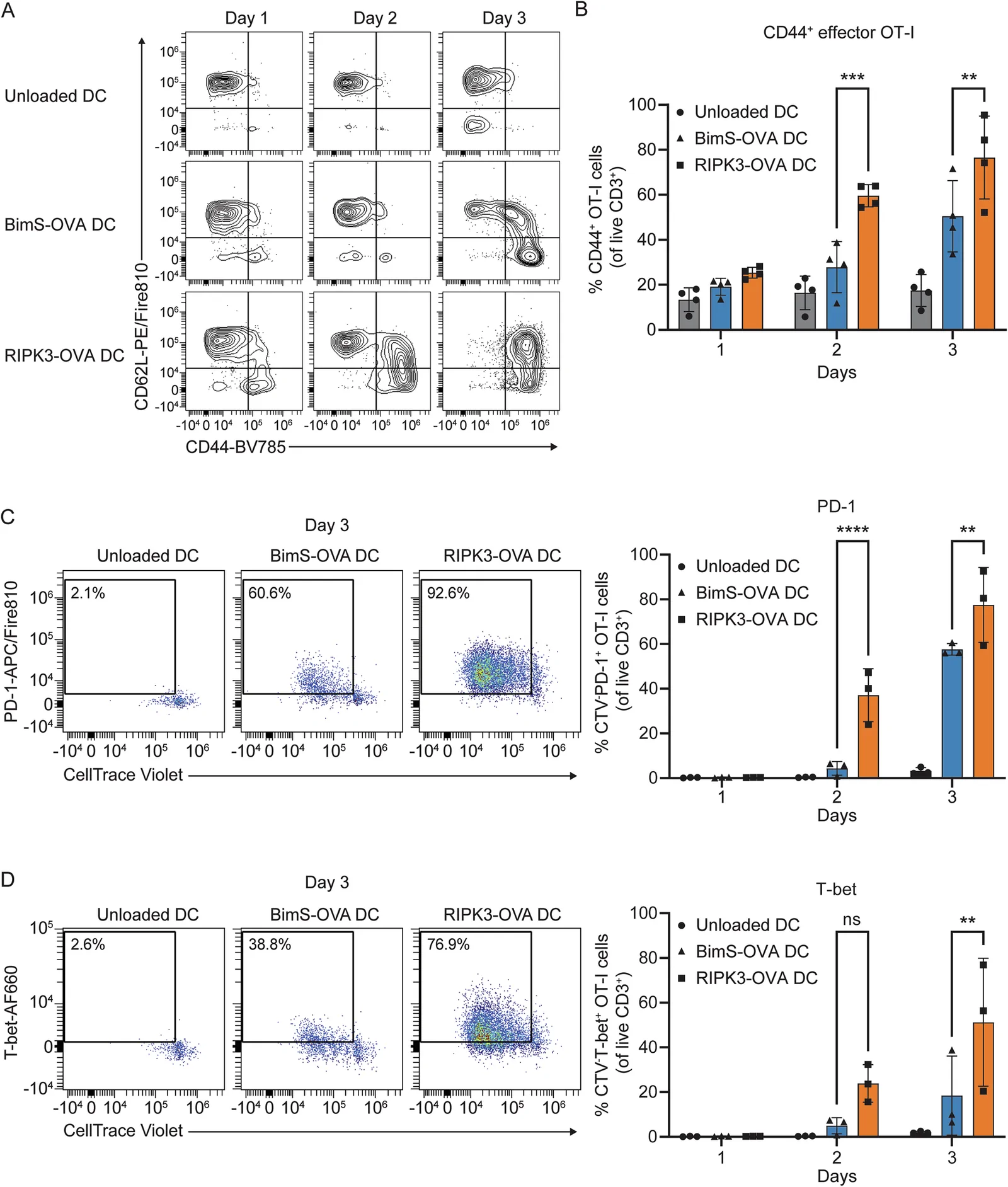
OVA-specific CD8^+^ T-cell effector differentiation induced by cDCs after phagocytosis of apoptotic or necroptotic TC-1-OVA cells. The cDC-OT-I co-culture platform was used as described in Fig. 2A and the phenotype of OT-I cells was read out at the indicated days by flow cytometry. **A, B** Representative contour plots depicting expression of CD62L and CD44 on OT-I cells **A** and bar graph showing frequency of CD44^+^ OT-I cells **B** at the indicated days after co-culture. **C** Representative flow cytometry scatterplot plot identifying the divided (CTV^-^) PD-1^+^ OT-I population (left panel) and bar graph showing frequency of CTV^-^PD-1^+^ OT-I cells (right panel) at the indicated days after co-culture. **D** Representative flow cytometry scatterplot plot indicating the CTV^-^T-bet^+^ OT-I population (left panel) and a bar graph showing frequency of CTV^-^T-bet^+^ OT-I cells (right panel) at the indicated days after co-culture. Means ± SD of 4 independent experiments in **B** and means ± SD of 3 independent experiments in **C** and **D** are shown. ns, not significant; **P < 0.01; ***P < 0.001; ****P < 0.0001; two-way ANOVA with Tukey’s multiple comparisons test.

We next determined the effects of apoptotic and necroptotic tumor cell death on effector differentiation of OT-I cells. Upon confrontation with OVA-loaded cDCs, OT-I T-cells acquired an activated effector phenotype, as identified by upregulation of CD44 and (partial) loss of CD62L expression (Fig. 3A). In the 3-day time frame, a proportion of OT-I cells stimulated by BimS-OVA DCs remained naive, whereas stimulation by RIPK3-OVA DCs resulted in a significantly larger proportion of CD44^+^ cells (Fig. 3A, B). Naive CD8^+^ T cells do not express programmed cell death protein 1 (PD-1), but it is rapidly upregulated upon TCR signaling [34]. Accordingly, stimulation by BimS-OVA DCs and RIPK3-OVA DCs increased the percentage of divided (CTV^-^)PD-1^+^ OT-I cells as compared to stimulation with unloaded cDCs (Fig. 3C). Moreover, the frequency of CTV^-^PD-1^+^ OT-I cells was significantly higher after stimulation by RIPK3-OVA DCs than BimS-OVA DCs (Fig. 3C), in agreement with a proportion of OT-I cells remaining naïve after stimulation with BimS-OVA DCs. The frequency of CTV^-^ OT-I cells expressing the transcription factor T-bet was higher after stimulation by RIPK3-OVA DCs than BimS-OVA DCs (Fig. 3D). This suggested better CTL effector differentiation of OT-I T cells after stimulation with RIPK3-OVA DCs, since T-bet is one of the key drivers of this process [35].

**Fig. 4:**
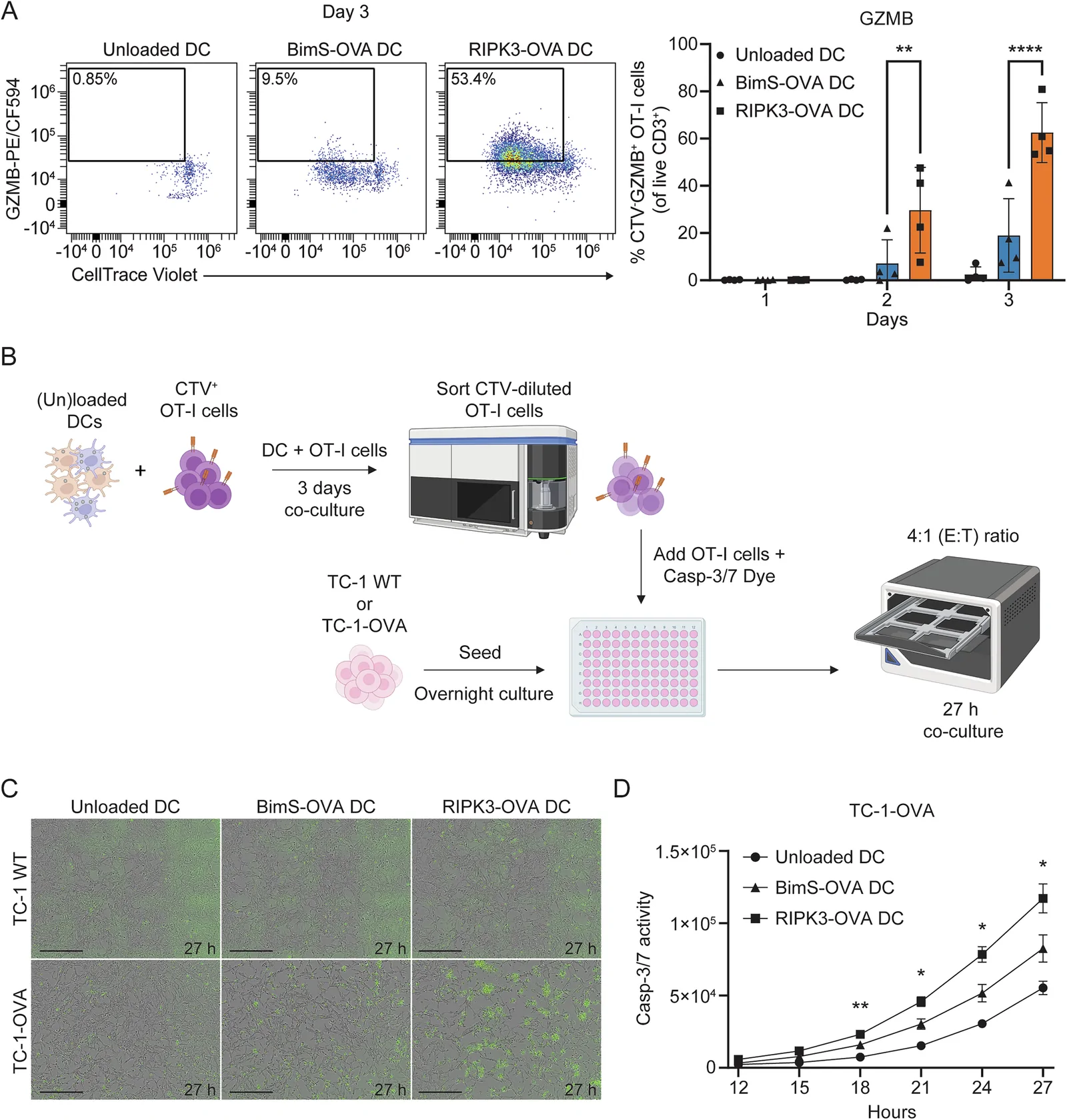
Cytotoxic function of OVA-specific CD8^+^ T-cells, as induced by cDCs after phagocytosis of apoptotic or necroptotic TC-1-OVA cells. The cDC-OT-I co-culture platform was used as described in Fig. 2A and phenotype and cytotoxic function of OT-I cells were read out. **A** Representative flow cytometry scatterplot indicating the CTV^-^GZMB^+^ OT-I population (left panel) and a bar graph showing the frequency of CTV^-^GZMB^+^ OT-I cells (right panel) at the indicated days after co-culture. **B** Experimental design of the killing assay. Created in BioRender. CTV-labeled OT-I cells and cDCs loaded with cell debris or unloaded were co-cultured at a 25:1 ratio and sorted OT-I cells as effectors (E) were incubated with TC-1 target (T) cells at a 4:1 ratio. **C, D** Representative brightfield images from Incucyte showing Caspase-3/7 activity by green fluorescence in TC-1 WT or TC-1-OVA target cells **C** and quantification of Caspase-3/7 activity in TC-1-OVA cells during the killing assay **D**. Scale bars in **C** represents 400 µm. Means ± SD of 4 independent experiments in **A** and means ± SD of 2 independent experiments in **D** are shown. Significance in **D** is indicated for BimS-OVA DCs vs. RIPK3-OVA DCs. *P < 0.05; **P < 0.01; ****P < 0.0001; two-way ANOVA with Tukey’s multiple comparisons test for **A** and two-way ANOVA for repeated measures with Tukey’s multiple comparisons test for **D**.

Indeed, the frequency of CTV^-^ OT-I cells expressing the key cytotoxic effector molecule granzyme B (GZMB) was much higher upon stimulation by RIPK3-OVA DCs compared to BimS-OVA DCs (Fig. 4A). To test killing capacity of OT-I cells, we first co-cultured CTV-labeled OT-I cells with cDCs as described in Fig. 2A and sorted CTV-diluted OT-I cells at day 3. These OT-I cells were tested for their capacity to kill either TC-1 WT or TC-1-OVA cells in an Incucyte assay, using Caspase-3/7 activity as read-out (Fig. 4B). TC-1-OVA cells, but not TC-1 WT cells showed Caspase-3/7 activity, indicating antigen-specific killing by OT-I cells (Fig. 4C). In line with higher GZMB levels (Fig. 4A), OT-I cells had a greater killing capacity after stimulation by RIPK3-OVA DCs than by BimS-OVA DCs (Fig. 4C, D). The collective data show that cDCs are better able to prime a CTL response after uptake of necroptotic tumor cells as compared to apoptotic tumor cells.

### Apoptotic cells debris is most efficiently phagocytosed by both cDC1s and cDC2s

**Fig. 5:**
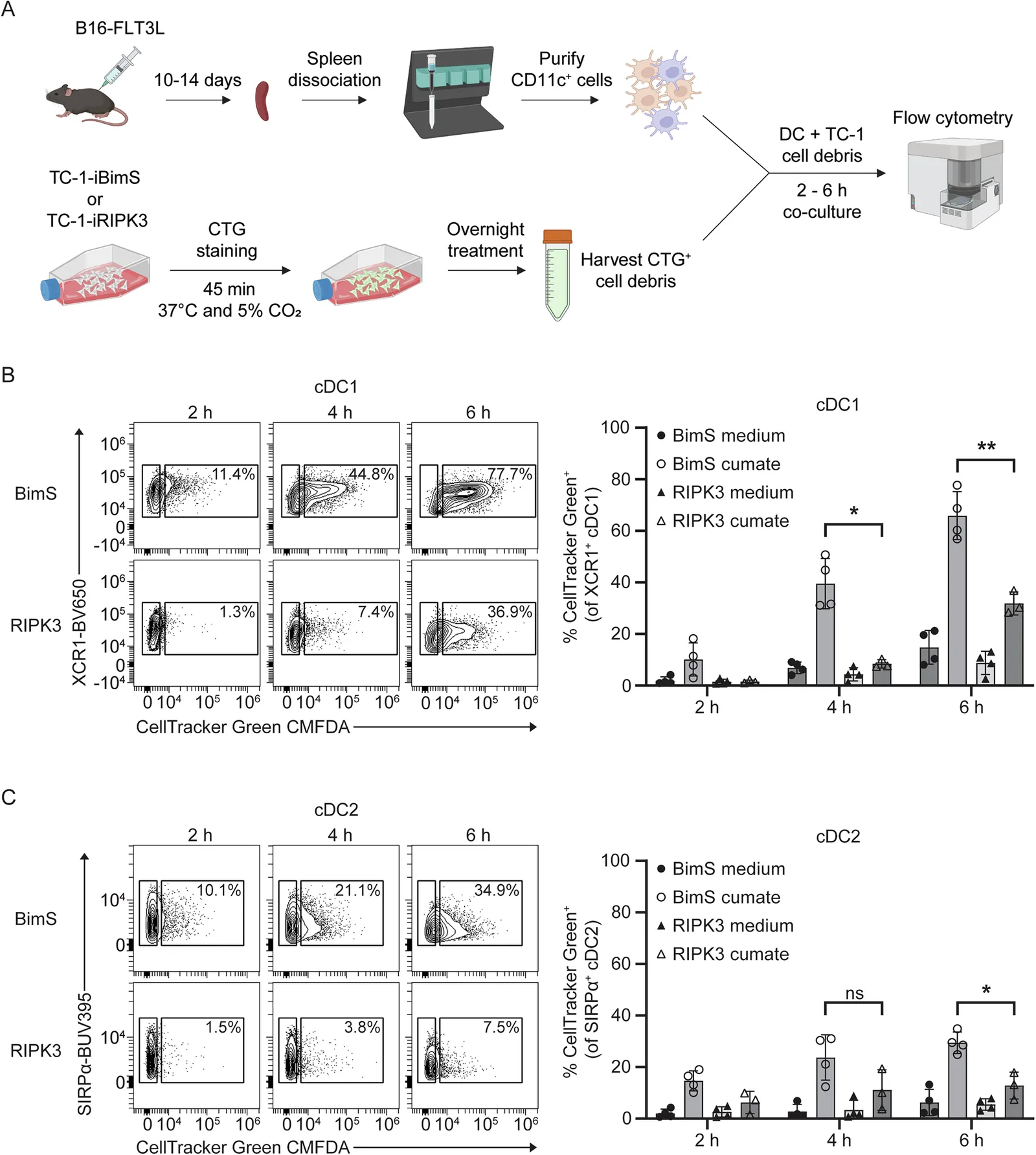
Phagocytosis of apoptotic or necroptotic TC-1 tumor cells by cDC1s and cDC2s. **A** Experimental design of the phagocytosis assay tracking CellTracker Green (CTG)^+^ dead tumor cells by flow cytometry. Created in BioRender. TC-1 cells were treated overnight with cumate and cell debris (medium) was added at a 1:1 ratio of plated TC-1 cells to cDCs. **B, C** Representative flow cytometry contour plots (left panels) and bar graphs (right panels) showing the frequency of CTG^+^ cDC1s **B** and cDC2s **C** present in the phagocytosis assay at the indicated time points. Means ± SD of 3 to 4 independent experiments are shown. ns, not significant; *P < 0.05; **P < 0.01; two-way ANOVA with Tukey’s multiple comparisons test.

To delineate why cDCs were more proficient in inducing a CTL response after uptake of necroptotic cell debris, we first examined how efficiently cDCs phagocytosed apoptotic versus necroptotic tumor cell debris. The cDCs were isolated as before and co-cultured with dead TC-1-iBimS and TC-1-iRIPK3 cells that had been labeled with CellTracker Green (CTG) CMFDA prior to cell death induction. This setup allowed us to track phagocytosis by flow cytometry as CTG fluorescence in both cDC1 and cDC2 subsets that were detected by specific cell surface markers (Fig. 5A). Total cDCs with a purity of 85% or higher were gated based on high MHC-II and CD11c expression and in this gate, cDC1s were discerned by XCR1 and CD8α co-expression and cDC2s by SIRPα and CD11b co-expression (Fig. S2). The frequency of CTG^+^ cDC1s (Fig. 5B) and cDC2s (Fig. 5C) increased over time after confrontation with either apoptotic or necroptotic cell debris, indicating phagocytic activity, which was lower for cDC2s than cDC1s, as expected [11]. Importantly, apoptotic debris was more efficiently phagocytosed than necroptotic debris by both cDC1s (Fig. 5B) and cDC2s (Fig. 5C). This difference could not be explained by a larger amount of debris being released after apoptosis, since uptake of apoptotic debris was still higher when cDCs were incubated with 2 to 4 times more necroptotic cells than apoptotic cells (Fig. S4A, B). We conclude therefore that both cDC1s and cDC2s phagocytose apoptotic tumor cell debris more efficiently than necroptotic tumor cell debris, with cDC1s being most proficient at phagocytosis.

### Maturation profiles of cDC1 and cDC2 differ after phagocytosis regardless of cell death mode

**Fig. 6:**
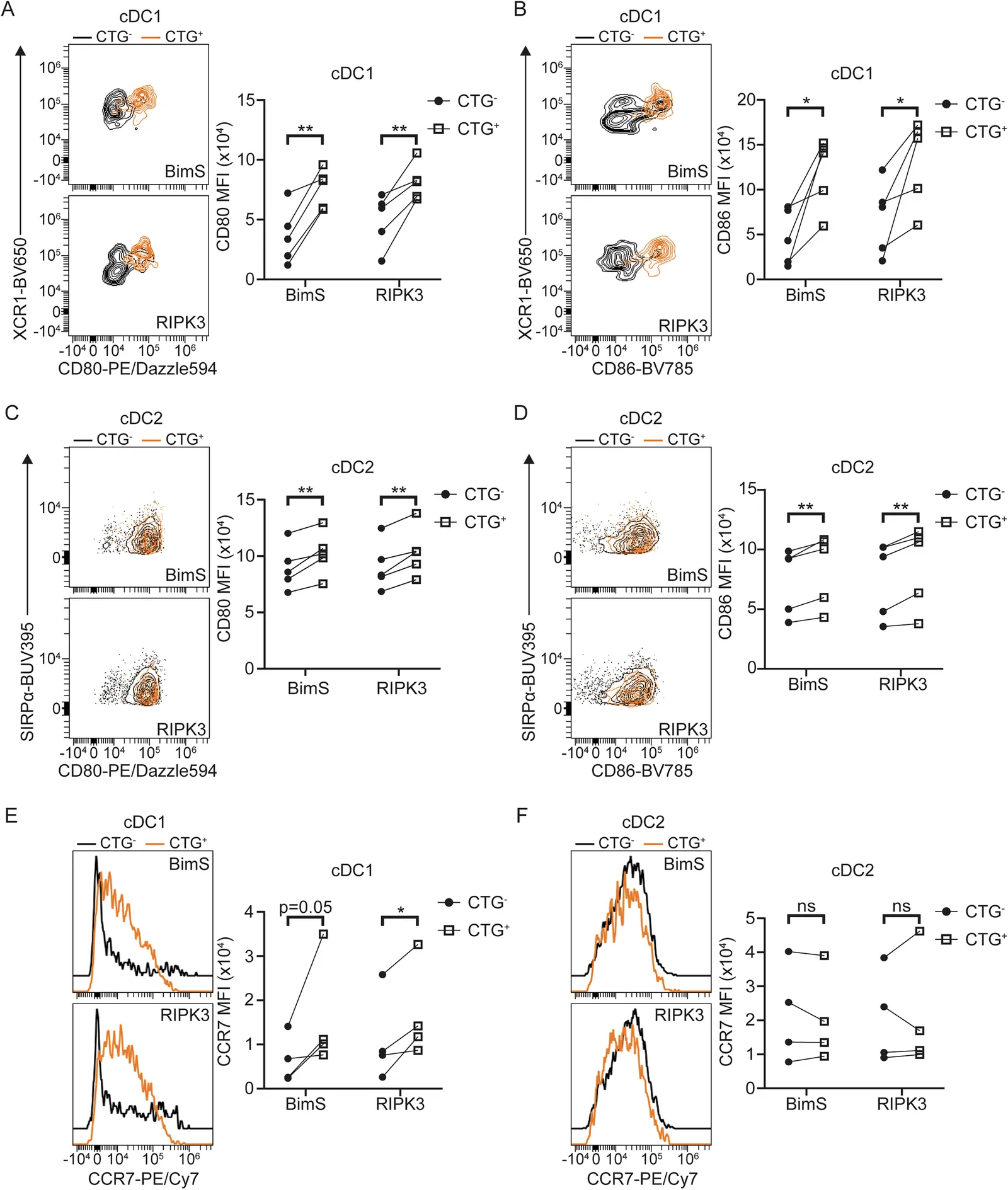
Cell surface phenotype of cDC1s and cDC2s after phagocytosis of apoptotic or necroptotic TC-1 tumor cells. The phagocytosis assay was carried out as described in Fig. 5A and cells in the culture were analyzed by flow cytometry. **A–D** Representative contour plots (left panels) and graphs showing the median fluorescence intensity (MFI) (right panels) of CD80 **A** and CD86 **B** in the CTG-negative and CTG positive populations in cDC1s **A, B** and cDC2s **C, D**. **E, F** Representative histograms (left panels) and graphs showing the MFI (right panels) of CCR7 in the CTG^-^ and CTG^+^ cDC1 **E** and cDC2 populations **F**. Each individual value within a graph belongs to an independent experiment. ns, not significant; *P < 0.05; **P < 0.01; one-tailed paired Student’s t-test.

To monitor maturation of cDC subsets, we analyzed expression of relevant cell surface markers by flow cytometry after 6 h of co-culture with CTG-labeled apoptotic or necroptotic tumor cells. Regardless of the mode of cell death, cDC1s that engulfed tumor cell debris according to CTG fluorescence showed similar increased expression of CD80 (Fig. 6A) and CD86 (Fig. 6B), the ligands of T-cell co-stimulatory receptor CD28 [34, 36]. cDC1s also had similar increased expression of CD40 (Fig. S5A), MHC-II (Fig. S5B) and PD-L1 (Fig. S5C), which all play an important role in cDC activation [37–39]. PD-L1 can bind to PD-1 that gives T cells an inhibitory signal, but it can also form a heterodimer with CD80 that selectively engages T-cell costimulatory receptor CD28 and relays signals important for cDC migration [39, 40]. cDC2s responded less strongly than cDC1s but acquired similar profiles after phagocytosing either apoptotic or necroptotic debris, upregulating CD80 (Fig. 6C), CD86 (Fig. 6D), CD40 (Fig. S5D), and MHC-II (Fig. S5E), but not PD-L1 (Fig. S5F). cDC1s (Fig. 6E), but not cDC2s (Fig. 6F) upregulated the chemokine receptor CCR7 after uptake of either type of tumor cell debris, which is critical for migration to dLNs [6]. These data show that concerning the markers measured, cDC1s and cDC2s show cell type-specific responses to uptake of dead cell debris, regardless of the mode of cell death.

### Uptake of necroptotic tumor cells induces a unique gene expression profile in cDC1s

**Fig. 7:**
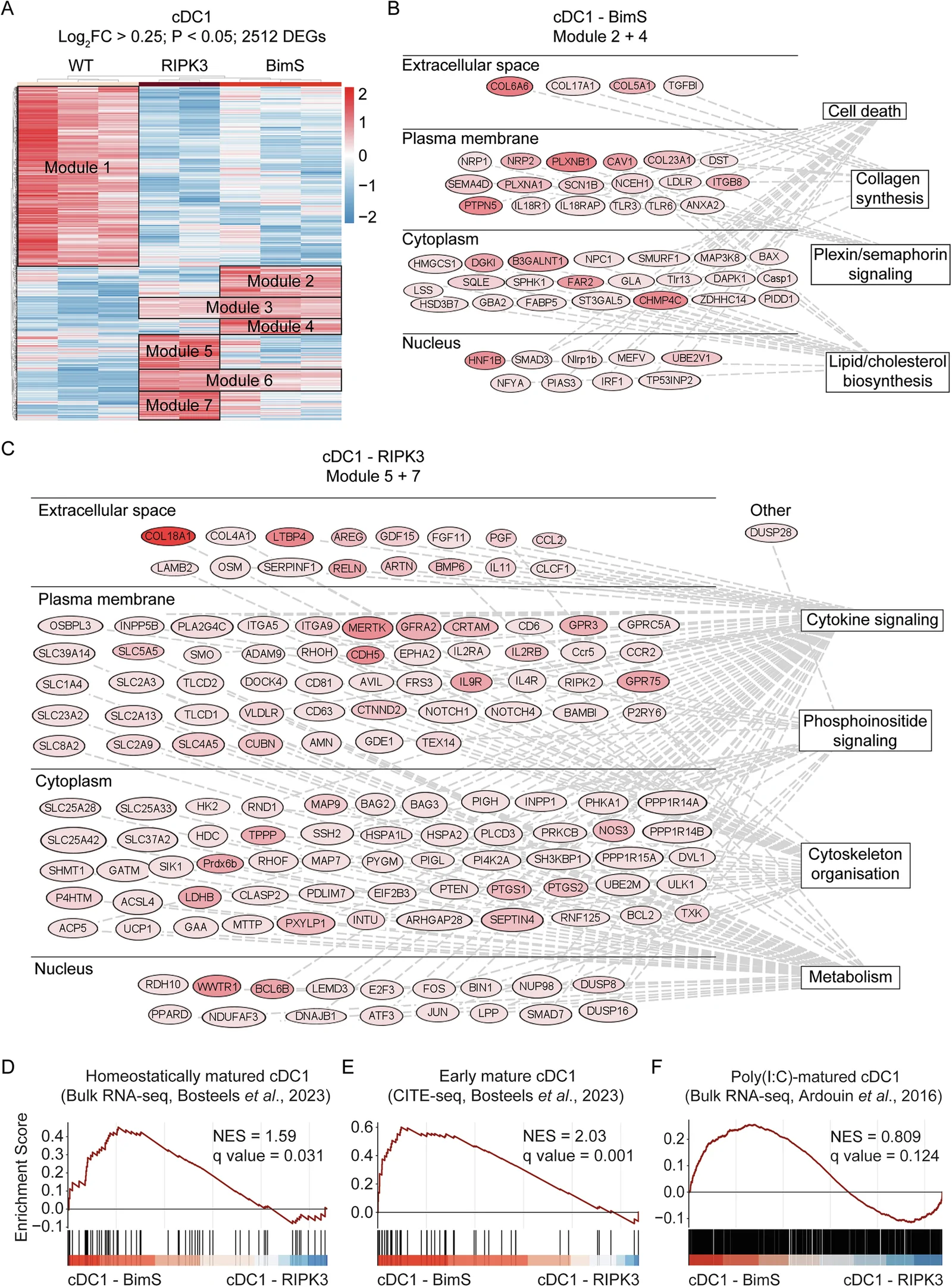
Gene expression profile of cDC1s after phagocytosis of apoptotic or necroptotic TC-1 tumor cells. The phagocytosis assay was carried out as described in Fig. 5A, after which cDC1s and cDC2s were flow cytometrically sorted, based on the gating strategy in Fig. S2, and each population was subjected to bulk transcriptome analysis. **A** Hierarchical clustering in heatmap of differentially expressed genes (DEGs) of sorted cDC1s incubated for 6 h with either medium from live TC-1 WT cells (WT), or necroptotic TC-1 tumor cell debris (RIPK3), or apoptotic TC-1 tumor cell debris (BimS). The gene list to generate the heatmap was based on a *P* value < 0.05 and a Log_2_FC > 0.25. Module 1: 1354 genes; module 2: 231 genes; module 3: 166 genes; module 4: 117 genes; module 5: 271 genes; module 6: 159 genes; module 7: 212 genes. **B, C** Ingenuity Pathway Analysis (IPA) of genes upregulated in BimS-specific modules 2 and 4 **B**, or in RIPK3-specific modules 5 and 7 **C**. Red color gradient indicates relative expression level. See Materials and Methods for details. **D, F** GSEA of published gene signatures of homeostatically matured [12] **D, E** or immunogenically matured [13] **F** splenic cDC1 in our transcriptomic data from cDC1s that encountered apoptotic or necroptotic tumor cell debris. Normalized Enrichment Score (NES) and q value of each analysis are shown. *q* value < 0.05 was considered statistically significant.

To deepen our understanding of the responses of cDC1s and cDC2s to apoptotic versus necroptotic tumor cell debris, we performed transcriptome analysis. The cDCs were prepared and exposed to tumor cell debris as described in Fig. 5A (without CTG staining), or to medium from wild-type (WT) TC-1 cells as a control. After 6 h, cDC1 and cDC2 populations were flow cytometrically sorted and subjected to bulk RNA sequencing. Comparative analysis of all samples revealed distinct gene expression profiles of cDC1s versus cDC2s, confirming their lineage identity **(**Fig. S6A, Supplementary Table 1), as hallmarked e.g. by *Clec9a*, *Xcr1, Wdfy4* and *Itgae* for cDC1s and *Sirpa* and *Clec10a* expression for cDC2s [7, 33, 41] (Fig. S6B). Among cDC1 samples, 2512 differentially expressed genes (DEGs) were found (Fig. 7A) and among cDC2 samples 511 DEGs (Fig. S7A). Certain genes were specifically upregulated in cDC2s co-cultured with either medium from living TC-1 WT cells (WT; module 1; 209 genes), necroptotic (RIPK3; module 2; 119 genes) or apoptotic tumor cell debris (BimS; module 4; 123 genes) (Fig. S7A, Supplementary Table 2). Gene Ontology (GO) analysis of module 1 genes revealed biological processes related to an IFN response (Fig. S7B). Both GO and Ingenuity Pathway Analysis (IPA) did not identify evident biological processes associated with gene modules 2 and 3 upregulated in RIPK3 samples (Fig. S7C) or gene modules 3 and 4 upregulated in BimS samples (Fig. S7D). These data underline that cDC1s rather than cDC2s are the main responders to dead cell debris.

The gene expression profiles of cDC1s co-cultured with apoptotic (BimS) or necroptotic (RIPK3) cell debris were different from each other and from cDC1s co-cultured with medium of WT TC-1 cells (Fig. 7A, Supplementary Table 3). The WT condition was characterized by upregulation of module 1 (1354 genes), the BimS condition by modules 2 and 4 (231 and 117 genes) and the RIPK3 condition by modules 5 and 7 (271 and 212 genes) and. Modules 3 and 6 (166 and 159 genes) were shared between BimS and RIPK3 conditions. As for cDC2s, GO analysis of module 1 genes revealed biological processes that were mainly related to cell division and response to IFN (Fig. S8A). Shared modules 3 and 6 contained genes involved in metabolism, endocytosis and cell death regulation (Fig. S8B). IPA of module 2 and 4 genes revealed a unique cDC1 state after uptake of apoptotic cell debris (Fig. 7B). We found upregulation of genes involved in cholesterol biosynthesis (e.g., *Hmgcs1*, *Npc1*, *Ldlr*, *Sqle*, *Lss*), which ties in with the recent finding that cholesterol biosynthesis characterizes homeostatic cDC1 maturation following uptake of apoptotic cells in vivo [12, 13, 42]. We also found upregulation of genes involved in chemorepulsion (e.g., *Sema4d*, *Nrp1*, *Nrp2*, *Plxna1*, *Plxnb1*), which may promote disruption of T cell-cDC communication. Genes linked to cell death (e.g., *Bax*, *Casp1, Nlrp1*) as well as genes that are involved in TGF-β signaling (e.g., *Itgb8*, *Col6a6*, *Col17a1*, *Col5a1*, *Tgfbi*, *Smad3, Smurf1*) were also upregulated. Gene Set Enrichment Analysis (GSEA) identified in these cDC1s the gene expression signature of homeostatically matured cDC1s that had taken up apoptotic cell debris in vivo [12] (Fig. 7D, E). This finding indicates that results from our in vitro system can be extrapolated to the in vivo setting.

IPA of module 5 and 7 genes related to uptake of necroptotic cell debris identified multiple secreted factors, as well as membrane, cytoplasmic and nuclear molecules involved in cytokine signaling (e.g., *Il11, Il2ra, Il2rb, Il4r, Il9*), phosphoinositide signaling (e.g. *Pten, Plcd3, Pi4k2a, Inpp1)*, cytoskeletal organization and cell adhesion (e.g., *Tppp*, *Ppp1r14a, Pppr14b*, *Rhof, Clasp2*, *Pdlim7, Itga5, Itga9*) and metabolism (Fig. 7C). The latter included many nutrient transporters (SLCs), among which the glucose transporter *Slc2a3* and key glycolytic enzyme *Hk2*. This gene expression pattern indicates an active cell state, with emphasis on cytoskeletal activity and functional differentiation [43]. The latter was underlined by upregulation of *Notch1*, *Notch4*, several Wnt pathway components (*Ctnnd2, Dvl, Wwtr1*) [44, 45], as well as diverse transcription factors/epigenetic regulators (e.g., *Bcl6B, Atf3*). Upregulation of *Bcl2* suggests improved cDC1 survival capacity [30]. The signature from cDC1s that had undergone immunogenic maturation after exposure to poly(I:C) in vivo [13] was not significantly enriched in cDC1s exposed to necroptotic tumor cell debris in vitro, as indicated (Fig. 7F). Since poly(I:C) is a PAMP that mimics infection, this result highlights the unique effect of necroptotic cell debris on cDC1s.

Importantly, differential expression of certain molecules in cDC1s that engulfed apoptotic versus necroptotic tumor cells as indicated by transcriptomics was also detected at the cell surface protein level by flow cytometry. cDC1s that engulfed apoptotic cells showed increased ITGB8 and SLAMF5 (CD84) protein levels, whereas SLAMF6 and NRP-1 levels did not differ between conditions (Fig. S9A). cDC1s that engulfed necroptotic tumor cells showed increased CRTAM (or CD355), IL-2Rβ (or CD122), and ITGA5 (or CD49e) protein levels, whereas CCR5 levels were similar between the two conditions (Fig. S9B). Of note, mRNA and protein expression generally do not correspond fully due to post-transcriptional and post-translational regulation and differential kinetics [46–48]. In conclusion, necroptotic tumor cells instruct a newly defined, active signaling and metabolic state within cDC1s that is likely responsible for the optimal capacity to induce CTL priming in vitro and may be extrapolated to in vivo settings, as shown for the cDC1 signature obtained after uptake of apoptotic cells.

## Discussion

We used cDCs generated from progenitors in vivo to determine the impact of apoptotic and necroptotic tumor cell death on cDC1 and cDC2 states and function. In our in vitro system, the dying TC-1 cells displayed the RCD-specific morphological features and died according to either apoptosis our necroptosis during the 20 h time frame of monitoring. The cDC1s and cDC2s used displayed characteristic gene expression patterns and functionality as described for ex vivo mouse and human equivalents [33]. In contrast, culturing bone marrow (BM) cells with GM-CSF generates primarily monocyte-derived DCs, which are different from cDCs [49–51]. We found that cDC1s more efficiently phagocytosed dead cell debris than cDC2s, regardless of the mode of cell death. This agrees with the facts that on cDC2s, SIRPα ligation by CD47 inhibits cytoskeletal rearrangements required for phagocytic function [9, 52], and that the cDC1 lineage is specialized in phagocytosing dead cell debris, as well as in antigen cross-presentation [7]. We found that cDC1s, but also cDC2s, phagocytosed apoptotic cells more efficiently than necroptotic cells, which might be explained by the nature of the cell debris. During apoptosis, cells dissociate into apoptotic bodies carrying various “eat-me” signals, including phosphatidylserine. Necroptotic cells also expose phosphatidylserine [53], but swell osmotically, then burst and largely remain as one entity, likely making phagocytosis more challenging. In accordance with their better phagocytic capacity, cDC1s showed more profound changes than cDC2s upon exposure to dead/dying cells in cell surface expression of CCR7, CD80, CD86 and CD40 and the overall transcriptome. Both cDC1s and cDC2s responded differently in terms of overall gene expression to medium of live tumor cells compared to dead tumor cells.

In accordance with literature data showing similar profiles of homeostatically and immunogenically matured cDC1s [6, 12, 13], CCR7, CD80, CD86 and CD40 cell surface levels on cDC1s did not differ significantly after exposure to apoptotic versus necroptotic tumor cell debris. However, transcriptome analysis clearly identified the differential response of cDC1s to apoptotic versus necroptotic cell debris. The uptake of apoptotic tumor cells in vitro induced a gene expression profile in cDC1s that shared key features with that of cDC1s that had homeostatically matured in vivo [12], arguing against a potential role of secondary necrosis on the cDC1 state. This finding indicates that our in vitro system can reveal physiologically relevant processes and mechanisms. A number of molecules upregulated in cDC1s at the mRNA level after phagocytosis of necroptotic versus apoptotic cells were confirmed at the cell surface protein level by flow cytometry, further validating our approach. Since transcriptome data on cDCs that have been exposed to necroptotically dying cells in vivo are not available, we could not validate in the same manner as for apoptotic cells the transcriptomic signature found in cDC1s responding to necroptotic cells. For these studies, induction of a defined RCD modality in vivo is preferred, as it excludes the risk that time consuming and physical handling of the dying cells prior to injection influences the outcome of the experiment. Our TC-1 tumor model had been engineered to induce apoptosis versus necroptosis in vitro as well as in vivo, but our attempts to induce gene expression by cumate injection in vivo were unfortunately not successful. This was likely due to the pharmacokinetics and pharmacodynamics of the inducer cumate, which are currently undefined. Recently, methodologies to induced the desired RCD in tumor cells have been published [27, 54], which will permit further examination of the impact of RCD modalities on cDC gene and protein expression and their capacity to induce T-cell responses.

cDC1s that had taken up apoptotic TC-1 cell debris in vitro showed a gene expression profile related to cholesterol metabolism, which is shared with homeostatically matured cDCs in vivo [12]. Upon uptake of apoptotic cells, cDC1s handle the overload of membrane lipid ingestion by strict regulation of lipid uptake and biosynthesis, specifically regarding cholesterol, as coordinated by the SREBP2 transcription factor [6, 12, 55]. We furthermore found indications for increased cellular repulsion and immunosuppressive activity of cDC1s that fit with tolerogenic properties of homeostatically matured cDC1s and will be of interest for follow-up functional studies. We newly define a gene expression profile of cDC1s responding to necroptotic tumor cells. This profile did not encompass the gene expression signature of immunogenic cDC1s responding to PAMPs, as defined by Ardouin et al. [13]. The functional processes activated in cDC1s after necroptotic cell ingestion according to their transcriptome agree with a more immunogenic state. This can be deduced from indications for functional differentiation, as well as increased glycolysis and cytoskeletal dynamics, tying in with evidence from BMDCs that glycolysis and migration are coupled processes [56, 57]. It will be of great interest to determine whether the gene expression signature we define here can be found back in ex vivo cDC1s and reliably reports phagocytosis of necroptotic tumor cells.

Functional annotation of expressed genes suggested an immunogenic state of the cDC1s, while cDC2s did not gain evident functionalities after phagocytosis of necroptotic cells according to their gene expression profile. Our collective data therefore strongly suggest that the more robust CTL response to cDC-based presentation of necroptotic cell debris as compared to apoptotic cell debris, was due to the gain in immunogenic properties of the cDC1s. The CD11c^+^ cell population that we used for our OT-I T cell response assays contained cDC1s and cDC2s in equal ratio and a small fraction of other immune cells, primarily B cells and some T cells, NK cells and pDCs (Fig. S10A). We confirmed that the cDC1s in this population were responsible for induction of the OT-I T cell response by purifying cDC1s on basis of XCR1 expression (Fig. S10B) and stimulating the OT-I cells side-by-side with the purified cDC1s or total CD11c^+^ cells loaded with apoptotic or necroptotic cell debris (Fig. S11). The assay system we use for immunogenicity of cDC populations after phagocytosing dead/dying cells can be extrapolated to the human setting by purifying cDCs ex vivo from blood and using T cells transduced with a specific TCR that can recognize defined tumor antigen in the context of a matched HLA molecule [58, 59].

Our data align with earlier studies demonstrating that necroptotic tumor cells are superior to apoptotic tumor cells in inducing a tumor-clearing CTL response in vivo [24–26], which relied on antigen presentation by cDC1s [25, 26]. NF-κB-dependent cytokine production in necroptotically dying tumor cells contributed to immunogenicity in this work, while another study found reliance on type I IFN production [28]. In general, transient IFN-I production is associated with immunogenicity in infection [13]. Also, cGAS-STING pathway activation leading to IFN-I production by tumor cells is an important immunogenic factor, as exemplified in the response to radiotherapy [60]. IFN-I is produced in response to a variety of DAMPs, in particular cytosolic DNA and RNA [2] and optimizes cDC1-mediated cross-presentation and T-cell priming by diverse mechanisms [4, 61]. However, in our setting, we did not find an IFN-response signature in the cDC1s that had phagocytosed necroptotic cells, suggesting that there are also other decisive elements dictating cDC1 immunogenicity.

Additional cDC1 gene expression studies delineating the impact of phagocytosing necroptotic cells in vivo in different tumor settings should help to clarify the decisive signals that lead to the immunogenic cDC1 state, the exact nature of this state and the mechanisms by which it overrules Treg activity to permit T-cell priming. It is attractive to purposely promote necroptotic cell death in cancer, but a limitation therein is the selective expression of RIPK3 and MLKL that constitute the necroptotic machinery [1, 20]. Importantly, tumor micro-environments including their immune cell constellations, differ not only between, but also within pathologically defined tumor types [62, 63] and are dictated by specific (oncogenic) signaling pathways in the cancer cells [64]. In particular, the propensity of a tumor to elicit a Treg response is decisive for spontaneous and therapy-induced T-cell priming. The lung cancer cell line TC-1 is a model for lymphocyte-depleted carcinomas in human. The CTL response to this tumor, as primed spontaneously and by radiotherapy in vivo, is severely constrained by simultaneous Treg priming [65]. Conventional radio- and chemotherapy likely kill tumor cells in vivo via a mixture of cell death modalities [15, 17], due to differential dosing, cellular heterogeneity and potential overload of phagocytes. It will be of interest to learn more about cDC1 states in tumors and tumor-draining LNs before and after conventional cancer therapies and correlate these with patient response. Such insights may help predict (potential) systemic immune responses and guide rational combination with (PD-1 targeting) immunotherapy.

## Materials and methods

### Cell lines

TC-1 tumor cells are C57BL/6-derived lung epithelial cells that express HPV16 antigens E6 and E7 and H-ras [29], and were from Leiden University Medical Center and tested negative for mycoplasma. TC-1 cells were cultured in Iscove’s Modified Dulbecco’s Medium (IMDM, Gibco) supplemented with 8% (v/v) heat-inactivated fetal bovine serum (FBS, Capricorn Scientific GmbH), 50 U/mL penicillin-streptomycin (Gibco), 1x MEM non-essential amino acids (Gibco) and 1 mM sodium pyruvate (Gibco). B16 mouse melanoma cells that were transduced in-house to express FLT3L and human embryonic kidney (HEK) 293 T cells were cultured in Dulbecco’s Modified Eagle’s Medium (DMEM, Gibco) supplemented with 8% (v/v) heat-inactivated FBS and 50 U/mL penicillin-streptomycin. Cell lines were cultured at 37 °C and 5% CO_2_.

### Plasmid generation

pCDH-EF1α-CymR-T2A-Puro SparQ and pCDH-CuO-MCS SparQ vectors were obtained from System Biosciences. pCDH-CuO-MCS was linearized with EcoRI-HF and BamHI-HF (New England Biolabs, NEB). BimS and RIPK3 coding sequences (*Mus musculus*) were obtained from GenBank (NCBI, NIH). The RIPK3 coding sequence was fused to a linker (amino acid sequence GSEGSEGSGS) followed by the 2L6HC3_13 homo-oligomer domain sequence [25] (kindly provided by Dr. A. Oberst, University of Washington, Seattle WA). A 5’ 25-base pair flanking region (GTCCAAGTTTGGTCTAGAGCTAGCG) and a 3’ 15- base pair flanking region (CGCGGCCGCGTCGAC) were added with homology to linearized pCDH-CuO-MCS. The complete DNA sequences were ordered as a gBlock gene fragment from Integrated DNA Technologies (IDT). The gBlocks were assembled into linearized pCDH-CuO-MCS, hereafter referred to as pCDH-CuO-BimS and pCDH-CuO-RIPK3 using NEBuilder HiFi DNA Assembly (NEB). Additionally, a DNA fragment containing the EF1α promoter, an internal ribosomal entry site (IRES) and the zsGreen coding sequence (referred to as EF1α-IRES-zsGreen) were inserted into pCDH-CuO-BimS and pCDH-CuO-RIPK3. The plasmids were linearized with NotI-HF (NEB), dephosphorylated using Antarctic Phosphatase (NEB) and purified by agarose gel extraction (ISOLATE II PCR and Gel Kit, Bioline). EF1α-IRES-zsGreen was obtained by PCR from pHIV-zsGreen (Addgene) with either sense primer 5’-ATGGAGAAGGCATTGAGCGCCGCTCTAGCGTGAGGC-3’ (for pCDH-CuO-BimS-zsGreen) or sense primer 5’-AACGCCAAAAGTGACTAACGCCGCTCTAGCGTGAGGC-3’ (for pCDH-CuO-RIPK3-zsGreen) and anti-sense primer 5’-CAGAGGTTGATTGTCGACGCCCGATCCTTAGGGCAAGG-3’, creating 20-base pair flanking regions homologous to NotI-linearized pCDH-CuO-BimS or pCDH-CuO-RIPK3. The PCR products were phosphorylated using T4 polynucleotide kinase (NEB) and assembled into pCDH-CuO-BimS or pCDH-CuO-RIPK3 with NEBuilder HiFi DNA Assembly, generating pCDH-CuO-BimS-zsGreen and pCDH-CuO-RIPK3-zsGreen. Full-length OVA coding sequence was obtained by PCR from an in-house plasmid and cloned into pCDH-EF1α using NEBuilder HiFi DNA Assembly. All plasmids were sequence-verified by Sanger sequencing and are available upon request.

### Lentivirus production and concentration

Third-generation lentiviruses were produced in HEK293T cells by polyethylenimine (PEI)-mediated transfection of packaging plasmids pCMV-VSVG, pMDLg-RRE, and pRSV-REV together with a transfer vector (pCDH-EF1α-CymR-T2A-Puro, pCDH-CuO-BimS-zsGreen, pCDH-CuO-RIPK3-zsGreen or pCDH-EF1α-OVA). The next day, medium was refreshed and at 48 h after transfection, lentivirus-containing medium was harvested. Cellular debris was removed by centrifugation (10 min, 700 *g*) and 0.45 µm filtration (Millex Low Protein Binding Durapore, Millipore) and virus was concentrated by sucrose-gradient centrifugation for 4 h at 10.000 *g* on a 10% (v/v) sucrose in 100 mM NaCl, 0.5 mM EDTA, 50 mM Tris-HCl pH 7.4 (1:4 v:v) [66]. Virus pellets were resuspended in ice-cold phosphate-buffered saline (PBS), aliquoted and stored at -80 °C. Lentivirus titers were determined by qPCR Lentivirus Titer Kit (Applied Biological Materials, abm).

### Generation of TC-1-iBimS and TC-1-iRIPK3 clones

TC-1 cells were reverse transduced with lentivirus encoding EF1α-CymR-T2A-Puro with a multiplicity of infection (MOI) of 0.1 in presence of 8 µg/mL polybrene. Medium was refreshed one day after transduction and cells were cultured for at least three days, after which transduced cells (referred to as TC-1-CymR) were selected with 4 µg/mL puromycin. Puromycin-resistant TC-1-CymR cells were reverse transduced with lentivirus encoding CuO-BimS-zsGreen or CuO-RIPK3-zsGreen with an MOI of 0.01 in the presence of 8 µg/mL polybrene. Medium was refreshed one day post-transduction and cells were cultured for at least one week, after which transduced cells (referred to as TC-1-iBimS and TC-1-iRIPK3) were flow cytometrically sorted based on zsGreen expression. Sorted TC-1-iBimS and TC-1-iRIPK3 cells were cultured for at least one week, after which they were sorted at 1 cell per well into 96 well plates with a Beckman Coulter CytoFLEX SRT Benchtop Cell Sorter to obtain clonal cell lines. Validated TC-1-iBimS and TC-1-iRIPK3 clones were reverse transduced with lentivirus encoding EF1α-OVA to obtain OVA-expressing cell lines TC-1-iBimS-OVA and TC-1-iRIPK3-OVA. OVA expression was validated by Western blot (Fig. S3C).

### Western blot

Cells were lysed for 20 min in ice-cold 1% NP-40 lysis buffer containing cOmplete Protease Inhibitor Cocktail (Roche), 150 mM NaCl and 50 mM Tris-HCl, pH 8.0 and lysate was centrifuged for 10 min at 13.000 *g* to remove nuclei. Protein concentration was determined with the Pierce BCA Protein Assay Kit (Thermo Fisher Scientific). Equal mass of protein per sample, taken up in NuPAGE LDS Sample buffer (Thermo Fisher Scientific) with dithiothreitol (87.5 mM) was denatured for 5 min at 95 °C and loaded onto a 4–12% NuPAGE Bis-Tris Protein Gel (Thermo Fisher Scientific), followed by protein separation in 1x NuPAGE MES SDS Running Buffer (Thermo Fisher Scientific). Proteins were transferred onto a 0.2 µm Nitrocellulose membrane (Bio-Rad) using the Trans-Blot Turbo Transfer System (Bio-Rad). The membrane was blocked with 10% (v/v) blocking buffer (Roche Western Blocking Reagent in Tris-buffered saline with Tween® 20 (TBS-T)) for 1 h at room temperature, incubated overnight at 4 °C with 16 µg/mL polyclonal rabbit anti-OVA antibody (Ab) (LSBio) and mouse anti-GAPDH Ab diluted in Western BLoT Immuno Booster 1 Solution (Takara), washed three times with TBS-T and incubated with goat anti-mouse IRDye^®^ 680LT (1:5000, LI-COR Biosciences) and goat anti-rabbit IRDye^®^ 800CW (1:5000, LI-COR Biosciences) for 1 h at room temperature in Western BLoT Immuno Booster 2 Solution (Takara). The membrane was washed three times and protein signal was directly visualized using the Odyssey CLx infrared imager (LI-COR Biosciences). Fluorescence intensity was measured using ImageJ software.

### Protein quantification assay

TC-1 WT, TC-1-iBimS-OVA, or TC-1-iRIPK3-OVA cells were cultured in serum-free Opti-MEM (Gibco) in the absence or presence of 800 µg/mL cumate (System Biosciences). After 24 h, the total medium was collected and directly subjected to the Pierce BCA Protein Assay Kit (Thermo Fisher Scientific), according to the manufacturer’s instructions, to determine the total protein concentration in each medium. Each biological replicate was performed in technical triplicates.

### Incucyte assays

TC-1-iBimS and TC-1-iRIPK3 cells were cultured with or without 800 µg/mL cumate (System Biosciences) in combination with either 0.5% (v/v) dimethyl sulfoxide (DMSO, vehicle control), or 50 µM pan-caspase inhibitor Q-VD-OPh (MedChemExpress), or 2 µM MLKL inhibitor GW806742x (MedChemExpress). Cell death and caspase activity were monitored by Incucyte® Cytotox Red Dye and Incucyte® Caspase-3/7 Red Dye. Dyes were purchased from Sartorius and diluted according to the manufacturer’s instructions. Data was acquired with an Incucyte S3 Live-Cell Imaging and Analysis System (Sartorius) and analyzed with Incucyte software.

### Mice

Eight-week-old female C57BL/6JRj were purchased from Janvier Laboratories (Le Genest-Saint-Isle, France) and female OT-I (C57BL/6-Tg(TcraTcrb)1100Mjb) mice on a CD45.1 background were bred in-house. Mice were housed in individually ventilated cages under specific pathogen-free conditions. Mice were euthanized by CO_2_ asphyxiation.

### Isolation of cDCs and OT-I T cells

For isolation of cDCs, C57BL/6JRj mice were anesthetized with isoflurane and injected s.c. in the flank with 1 × 10^6^ B16-FLT3L cells in 100 µL Hank’s Balanced Salt Solution (HBSS, Gibco). After 10 to 14 days when tumors were visible and palpable, mice were sacrificed and spleens were isolated. Spleens were cut into small pieces with a scalpel and incubated with 100 µg/mL Liberase TL Research Grade (Roche) and 10 µg/mL DNaseI (Roche) in serum-free DMEM for 30 min in a shaking 37 °C water bath. Spleens were washed in IMDM-FBS, dispersed through a 70 µm nylon cell strainer (Falcon) and the remaining cell suspension was centrifuged for 5 min at 500 *g*. Splenocytes were resuspended in 1x RBC Lysis Buffer (ChemCruz, Santa Cruz Biotechnology) and incubated for 1 min at room temperature to remove red blood cells. IMDM-FBS was added, splenocytes were centrifuged for 5 min at 400 *g* and the pellet was resuspended in IMDM-FBS. CD11c^+^ cells were isolated from splenocytes by positive selection using CD11c MicroBeads UltraPure (Miltenyi Biotec), according to the manufacturer’s instructions. A CD11c^+^ cell purity of 85% or higher was used for experiments. This cell population contained cDC1s and cDC2s as defined by discerning markers [33] (Fig. S2) in an about 1:1 ratio. For the experiment shown in Fig. S11, XCR1^+^ cells were isolated to 95% purity or higher with the anti-XCR1 MicroBead Kit (Miltenyi Biotec) (Fig. S10). OT-I cells were isolated from spleens of female OT-I*CD45.1 mice. Splenocytes were isolated and resuspended in IMDM-FBS, after which OT-I cells were isolated by negative selection using the CD8α^+^ T Cell Isolation Kit (Miltenyi Biotec) according to the manufacturer’s instructions. OT-I cells were labeled with 5 µM CellTrace Violet (CTV) Cell Proliferation Kit (Thermo Fisher Scientific) according to the manufacturer’s instructions. For each experiment, spleens from at least two mice were pooled to account for possible inter-mouse variability.

### Phagocytosis

TC-1-iBimS and TC-1-iRIPK3 cells were stained with 1 µM CellTracker Green (CTG) CMFDA dye (Thermo Fisher Scientific) in serum-free IMDM for 30 min at 37 °C and 5% CO_2_, washed twice in culture medium and cultured. CFMDA is cell permeable chloromethyl derivative of fluorescein diacetate that crosslinks covalently with any available intracellular thiol side chains. One day later, cell death was induced by culture with 800 µg/mL cumate (System Biosciences) for at least 24 h. Next, the medium overlaying the TC-1 cells with all its contents was collected, thereby harvesting the dead or dying cells, cell debris and soluble material. Generally, cell death induction was very efficient and very few live and adherent TC-1 cells remained in the culture dish. The medium from the TC-1 cells was not centrifuged, but added directly to CD11c^+^ cells at a ratio of 1:1 (i.e. 50.000 CD11c^+^ cells incubated with medium from 50.000 TC-1 cells). To improve CD11c^+^ cell survival, 2 ng/mL recombinant mouse GM-CSF (carrier-free, BioLegend) was added to the culture. After co-culture, CD11c^+^ cells were incubated with CD16/CD32 Fc Block (1:50, clone 2.4G2, BD Biosciences #553141) for 10 min on ice, followed by cell surface staining for flow cytometry.

### Co-culture of cDCs and OT-I cells

TC-1-iBimS-OVA and TC-1-iRIPK3-OVA cells were cultured in presence of 800 µg/mL water-soluble cumate (System Biosciences) for 24 h to induce apoptosis or necroptosis, after which the medium from the dead/dying cells was collected as outlined in the preceding section “Phagocytosis”. Per experimental condition, 2 × 10^3^ CD11c^+^ cells were loaded with medium from 2 × 10^3^ apoptotic TC-1-iBimS-OVA or necroptotic TC-1-iRIPK3-OVA cells for 6 h at 37 °C and 5% CO_2_, in the presence of 2 ng/mL mouse recombinant GM-CSF [67] (carrier-free, BioLegend). After loading, CD11c^+^ cells were washed with IMDM-FBS by centrifugation (5 min, 500 *g*) and 5 ×10^4^ CTV-labeled OT-I cells were added to 2 ×10^3^ CD11c^+^ cells (OT-I-cDC ratio 25:1). OT-I cells were cultured for the indicated number of days in the presence of 2 ng/mL recombinant mouse IL-7 (carrier-free, BioLegend) and stained for flow cytometry. For the experiment outlined in Fig. S11, XCR1^+^ cells were used, next to CD11c^+^ cells.

### Flow cytometry

All cell surface Ab stainings were performed in ice-cold PBS supplemented with 2% FBS for 30 min on ice in the dark, except for CCR5 and CCR7 which were stained for 30 min at 37 °C. Zombie UV Fixable Viability Kit (1:500, BioLegend #423107) was added to all cell surface Ab staining mixes to exclude dead cells. Next, cells were washed with ice-cold PBS containing 2% FBS by centrifugation (5 min, 500 *g*), fixed and permeabilized with eBioscience Foxp3/Transcription Factor Staining Buffer Set (Thermo Fisher Scientific) according to the manufacturer’s instructions. Intracellular Ab staining of OT-I cells was done for 45 min on ice in the dark. Finally, cells were washed with 1x Permeabilization Buffer, centrifuged for 5 min at 500 *g* and collected in ice-cold PBS containing 2% FBS. All antibodies used are provided in Supplementary Table 4. Cells were analyzed on a Cytek 5-laser Aurora spectral flow cytometer. Data was analyzed with FlowJo V10 (BD Biosciences) and OMIQ software (Dotmatics).

### Bulk RNA sequencing

TC-1 WT, TC-1-iBimS, and TC-1-iRIPK3 cells were incubated with 800 µg/mL cumate (System Biosciences) for 24 h, after which cell medium was collected. CD11c^+^ cells were cultured for 6 h with this medium at a 1:1 ratio as outlined above. Next, cDC1s and cDC2s were sorted based on the gating strategy in Fig. S2, using a BD FACSAria™ III Cell Sorter. Cells were centrifuged for 5 min at 500 *g* and the cell pellet was resuspended in 350 µL RLT buffer (Qiagen), snap-frozen in liquid nitrogen and stored at -80 °C until sequencing procedure.

Libraries were prepared using the xGen™ RNA Library Prep (Integrated DNA Technologies) with an “on-bead” poly-A enrichment using the NEBNext® Poly(A) mRNA Magnetic Isolation Module (NEB). Sequencing was performed on an Illumina platform (NovaSeq 6000 Sequencing System) to generate 2 × 54 bp paired-end reads, targeting a depth of 20 million paired-end reads per sample. Seqpurge trimmed reads were aligned to the *Mus musculus* GRCm39 reference genome (annotation source: Ensembl v112) using STAR (v2.7.10a); key parameters: ‘--runRNGseed 12031 --quantMode GeneCounts --outSAMattributes NH HI NM MD AS --sjdbOverhang 53 --alignSJDBoverhangMin 1 -sjdbGTFfile ${gtf}’, achieving an average mapping rate of 93%. Gene/transcript quantification was performed using gensum (version: 0.2.1).

RNAseq data were transformed as log₂(count + 1), and only mRNAs with nonzero counts in at least half of the samples were retained for downstream analyses. Differential gene expression analysis was performed with R package limma (v3.58.1) based on the raw count matrix. A linear model was fit via lmFit(expr_mat, design), contrasts were specified with contrasts.fit, and statistics were computed using eBayes(trend=TRUE, robust=TRUE); P values were adjusted by the Benjamini–Hochberg method and genes with |log₂ FC | > 0.5 and a P value < 0.05 were considered significant. Heatmaps were generated with the R package pheatmap (v1.0.12) using Euclidean distance and complete linkage clustering on both rows (genes) and columns (samples) with row-wise Z-score normalization (scale = “row”). Volcano plot was generated in ggplot2 (v3.4.1) with log₂ FC on the x-axis and –log₁₀(p value) on the y-axis. Upregulated and downregulated genes were colored differently. Enrichment analysis of differentially expressed genes was performed using the R package clusterProfiler (v4.6.0). Gene Ontology biological processes were identified using the STRING database (https://string-db.org/) with a similarity filter cutoff of >=0.4. Ingenuity Pathway Analysis (IPA) software (Qiagen) provided biological processes with overlapping annotations and genes, from which we curated gene lists that were uploaded to IPA My Pathway. Genes were ordered based on subcellular localization and we annotated each group of genes based on the biological process they were linked to according to IPA.

### Statistical analysis

Data visualization and statistical analyses were done using GraphPad Prism v10.2.3 (Dotmatics). Data were tested for normal distribution and equal variance before performing statistical analyses. Comparisons between two groups were assessed by a Student’s t-test, and comparisons with more than two groups were assessed by either a one-way ANOVA (for one independent variable) or two-way ANOVA (for two independent variables) with Tukey’s multiple comparisons test. Data are presented as mean ± SD from biological replicates, as indicated in the figure legends. Sample size was based on availability, and for experiments with primary immune cells, spleens from at least two mice were pooled to account for variability between mice. A *P* value < 0.05 was considered statistically significant; *P < 0.05, **P < 0.01, ***P < 0.001, ****P < 0.0001.

## Supplementary information

Supplementary Information

Movie 1.

Movie 2

Movie 3

Movie 4

Movie 5

Dataset 1

Dataset 2

Dataset 3

Dataset 4

## Data Availability

Bulk RNAseq data are available on NCBI GEO under accession number GSE314783. Materials and raw data will be provided upon reasonable request.

## References

[1] Meier P, Legrand AJ, Adam D, Silke J. Immunogenic cell death in cancer: targeting necroptosis to induce antitumour immunity. Nat Rev Cancer. 2024;24:299–315. 38454135 10.1038/s41568-024-00674-x

[2] Galluzzi L, Vitale I, Warren S, Adjemian S, Agostinis P, Martinez AB, et al. Consensus guidelines for the definition, detection and interpretation of immunogenic cell death. J Immunother Cancer. 2020;8:e000337. 10.1136/jitc-2019-000337 PMC7064135 32209603

[3] Mellman I, Chen DS, Powles T, Turley SJ. The cancer-immunity cycle: Indication, genotype, and immunotype. Immunity. 2023;56:2188–205. 37820582 10.1016/j.immuni.2023.09.011

[4] Pittet MJ, Di Pilato M, Garris C, Mempel TR. Dendritic cells as shepherds of T cell immunity in cancer. Immunity. 2023;56:2218–30. 37708889 10.1016/j.immuni.2023.08.014 PMC10591862

[5] Borst J, Ahrends T, Babala N, Melief CJM, Kastenmuller W. CD4(+) T cell help in cancer immunology and immunotherapy. Nat Rev Immunol. 2018;18:635–47. 30057419 10.1038/s41577-018-0044-0

[6] Janssens S, Rennen S, Agostinis P. Decoding immunogenic cell death from a dendritic cell perspective. Immunol Rev. 2024;321:350–70. 38093416 10.1111/imr.13301

[7] Ohara RA, Murphy KM. Recent progress in type 1 classical dendritic cell cross-presentation - cytosolic, vacuolar, or both? Curr Opin Immunol. 2023;83:102350. 37276818 10.1016/j.coi.2023.102350 PMC12013855

[8] Eisenbarth SC. Dendritic cell subsets in T cell programming: location dictates function. Nat Rev Immunol. 2019;19:89–103. 30464294 10.1038/s41577-018-0088-1 PMC7755085

[9] Logtenberg MEW, Scheeren FA, Schumacher TN. The CD47-SIRPalpha Immune Checkpoint. Immunity. 2020;52:742–52. 32433947 10.1016/j.immuni.2020.04.011 PMC7340539

[10] van Duijn A, Van der Burg SH, Scheeren FA. CD47/SIRPalpha axis: bridging innate and adaptive immunity. J Immunother Cancer. 2022;10:e004589. 10.1136/jitc-2022-004589 PMC9280883 35831032

[11] Cabeza-Cabrerizo M, Cardoso A, Minutti CM, Pereira da Costa M, Reis e Sousa C. Dendritic Cells Revisited. Annu Rev Immunol. 2021;39:131–66. 33481643 10.1146/annurev-immunol-061020-053707

[12] Bosteels V, Marechal S, De Nolf C, Rennen S, Maelfait J, Tavernier SJ, et al. LXR signaling controls homeostatic dendritic cell maturation. Sci Immunol. 2023;8:eadd3955. 37172103 10.1126/sciimmunol.add3955

[13] Ardouin L, Luche H, Chelbi R, Carpentier S, Shawket A, Montanana Sanchis F, et al. Broad and Largely Concordant Molecular Changes Characterize Tolerogenic and Immunogenic Dendritic Cell Maturation in Thymus and Periphery. Immunity. 2016;45:305–18. 27533013 10.1016/j.immuni.2016.07.019

[14] Liu Z, Gerner MY, Van Panhuys N, Levine AG, Rudensky AY, Germain RN. Immune homeostasis enforced by co-localized effector and regulatory T cells. Nature. 2015;528:225–30. 26605524 10.1038/nature16169 PMC4702500

[15] Yuan J, Ofengeim D. A guide to cell death pathways. Nat Rev Mol Cell Biol. 2024;25:379–95. 38110635 10.1038/s41580-023-00689-6

[16] Chen R, Zou J, Liu J, Kang R, Tang D. DAMPs in the immunogenicity of cell death. Mol Cell. 2025;85:3874–89. 41106375 10.1016/j.molcel.2025.09.007

[17] Hanggi K, Ruffell B. Cell death, therapeutics, and the immune response in cancer. Trends Cancer. 2023;9:381–96. 36841748 10.1016/j.trecan.2023.02.001 PMC10121860

[18] Kroemer G, Galassi C, Zitvogel L, Galluzzi L. Immunogenic cell stress and death. Nat Immunol. 2022;23:487–500. 35145297 10.1038/s41590-022-01132-2

[19] Tanzer MC, Frauenstein A, Stafford CA, Phulphagar K, Mann M, Meissner F. Quantitative and Dynamic Catalogs of Proteins Released during Apoptotic and Necroptotic Cell Death. Cell Rep. 2020;30:1260–70.e5. 31995763 10.1016/j.celrep.2019.12.079

[20] Vince JE, Davidson NM, Tanzer MC. Necroptotic cell death consequences and disease relevance. Nat Immunol. 2025;26:1863–76. 41094200 10.1038/s41590-025-02298-1

[21] Ovcinnikovs V, Ross EM, Petersone L, Edner NM, Heuts F, Ntavli E, et al. CTLA-4-mediated transendocytosis of costimulatory molecules primarily targets migratory dendritic cells. Sci Immunol. 2019;4:eaaw0902. 10.1126/sciimmunol.aaw0902 PMC6570622 31152091

[22] Sun L, Wang H, Wang Z, He S, Chen S, Liao D, et al. Mixed lineage kinase domain-like protein mediates necrosis signaling downstream of RIP3 kinase. Cell. 2012;148:213–27. 22265413 10.1016/j.cell.2011.11.031

[23] Cho YS, Challa S, Moquin D, Genga R, Ray TD, Guildford M, et al. Phosphorylation-driven assembly of the RIP1-RIP3 complex regulates programmed necrosis and virus-induced inflammation. Cell. 2009;137:1112–23. 19524513 10.1016/j.cell.2009.05.037 PMC2727676

[24] Aaes TL, Kaczmarek A, Delvaeye T, De Craene B, De Koker S, Heyndrickx L, et al. Vaccination with Necroptotic Cancer Cells Induces Efficient Anti-tumor Immunity. Cell Rep. 2016;15:274–87. 27050509 10.1016/j.celrep.2016.03.037

[25] Snyder AG, Hubbard NW, Messmer MN, Kofman SB, Hagan CE, Orozco SL, et al. Intratumoral activation of the necroptotic pathway components RIPK1 and RIPK3 potentiates antitumor immunity. Sci Immunol. 2019;4:eaaw2004. 10.1126/sciimmunol.aaw2004 PMC6831211 31227597

[26] Yatim N, Jusforgues-Saklani H, Orozco S, Schulz O, Barreira da Silva R, Reis e Sousa C, et al. RIPK1 and NF-kappaB signaling in dying cells determines cross-priming of CD8(+) T cells. Science. 2015;350:328–34. 26405229 10.1126/science.aad0395 PMC4651449

[27] Chen KS, Manoury-Battais S, Kanaya N, Vogiatzi I, Borges P, Kruize SJ, et al. An inducible RIPK3-driven necroptotic system enhances cancer cell-based immunotherapy and ensures safety. J Clin Invest. 2024;135:e181143. 10.1172/JCI181143 PMC11735097 39560995

[28] Rucker AJ, Park CS, Li QJ, Moseman EA, Chan FK. Necroptosis stimulates interferon-mediated protective anti-tumor immunity. Cell Death Dis. 2024;15:403. 38858387 10.1038/s41419-024-06801-8 PMC11164861

[29] Lin KY, Guarnieri FG, Staveley-O’Carroll KF, Levitsky HI, August JT, Pardoll DM, et al. Treatment of established tumors with a novel vaccine that enhances major histocompatibility class II presentation of tumor antigen. Cancer Res. 1996;56:21–6. 8548765

[30] Bedoui S, Herold MJ, Strasser A. Emerging connectivity of programmed cell death pathways and its physiological implications. Nat Rev Mol Cell Biol. 2020;21:678–95. 32873928 10.1038/s41580-020-0270-8

[31] Mullick A, Xu Y, Warren R, Koutroumanis M, Guilbault C, Broussau S, et al. The cumate gene-switch: a system for regulated expression in mammalian cells. BMC Biotechnol. 2006;6:43. 17083727 10.1186/1472-6750-6-43 PMC1654148

[32] Mach N, Gillessen S, Wilson SB, Sheehan C, Mihm M, Dranoff G. Differences in dendritic cells stimulated by tumors engineered to secrete granulocyte-macrophage colony-stimulating factor or flt3-ligand. Cancer Res. 2000;60:3239–46. 10866317

[33] Guilliams M, Dutertre CA, Scott CL, McGovern N, Sichien D, Chakarov S, et al. Unsupervised High-Dimensional Analysis Aligns Dendritic Cells across Tissues and Species. Immunity. 2016;45:669–84. 27637149 10.1016/j.immuni.2016.08.015 PMC5040826

[34] Burke KP, Chaudhri A, Freeman GJ, Sharpe AH. The B7:CD28 family and friends: Unraveling coinhibitory interactions. Immunity. 2024;57:223–44. 38354702 10.1016/j.immuni.2024.01.013 PMC10889489

[35] Sullivan BM, Juedes A, Szabo SJ, von Herrath M, Glimcher LH. Antigen-driven effector CD8 T cell function regulated by T-bet. Proc Natl Acad Sci USA. 2003;100:15818–23. 14673093 10.1073/pnas.2636938100 PMC307651

[36] Acuto O, Michel F. CD28-mediated co-stimulation: a quantitative support for TCR signalling. Nat Rev Immunol. 2003;3:939–51. 14647476 10.1038/nri1248

[37] Wu R, Ohara RA, Jo S, Liu TT, Ferris ST, Ou F, et al. Mechanisms of CD40-dependent cDC1 licensing beyond costimulation. Nat Immunol. 2022;23:1536–50. 36271147 10.1038/s41590-022-01324-w PMC9896965

[38] Gressier E, Schulte-Schrepping J, Petrov L, Brumhard S, Stubbemann P, Hiller A, et al. CD4(+) T cell calibration of antigen-presenting cells optimizes antiviral CD8(+) T cell immunity. Nat Immunol. 2023;24:979–90. 37188942 10.1038/s41590-023-01517-x

[39] Kantheti U, Forward TS, Lucas ED, Schafer JB, Tamburini PJ, Burchill MA, et al. PD-L1-CD80 interactions are required for intracellular signaling necessary for dendritic cell migration. Sci Adv. 2025;11:eadt3044. 39879305 10.1126/sciadv.adt3044 PMC11777207

[40] Zhao Y, Lee CK, Lin CH, Gassen RB, Xu X, Huang Z, et al. PD-L1:CD80 Cis-Heterodimer Triggers the Co-stimulatory Receptor CD28 While Repressing the Inhibitory PD-1 and CTLA-4 Pathways. Immunity. 2019;51:1059–73.e9. 31757674 10.1016/j.immuni.2019.11.003 PMC6935268

[41] Brown CC, Gudjonson H, Pritykin Y, Deep D, Lavallee VP, Mendoza A, et al. Transcriptional Basis of Mouse and Human Dendritic Cell Heterogeneity. Cell. 2019;179:846–63.e24. 31668803 10.1016/j.cell.2019.09.035 PMC6838684

[42] Belabed M, Park MD, Blouin CM, Balan S, Moon CY, Freed G, et al. Cholesterol mobilization regulates dendritic cell maturation and the immunogenic response to cancer. Nat Immunol. 2025;26:188–99. 39838105 10.1038/s41590-024-02065-8

[43] Hou X, Chen Y, Carrillo ND, Cryns VL, Anderson RA, Sun J, et al. Phosphoinositide signaling at the cytoskeleton in the regulation of cell dynamics. Cell Death Dis. 2025;16:296. 40229242 10.1038/s41419-025-07616-x PMC11997203

[44] Cheng P, Zhou J, Gabrilovich D. Regulation of dendritic cell differentiation and function by Notch and Wnt pathways. Immunol Rev. 2010;234:105–19. 20193015 10.1111/j.0105-2896.2009.00871.x

[45] Zhou J, Cheng P, Youn JI, Cotter MJ, Gabrilovich DI. Notch and wingless signaling cooperate in regulation of dendritic cell differentiation. Immunity. 2009;30:845–59. 19523851 10.1016/j.immuni.2009.03.021 PMC2700307

[46] Cuadrado E, van den Biggelaar M, de Kivit S, Chen YY, Slot M, Doubal I, et al. Proteomic Analyses of Human Regulatory T Cells Reveal Adaptations in Signaling Pathways that Protect Cellular Identity. Immunity. 2018;48:1046–59.e6. 29752063 10.1016/j.immuni.2018.04.008

[47] Jovanovic M, Rooney MS, Mertins P, Przybylski D, Chevrier N, Satija R, et al. Immunogenetics. Dynamic profiling of the protein life cycle in response to pathogens. Science. 2015;347:1259038. 25745177 10.1126/science.1259038 PMC4506746

[48] Vogel C, Marcotte EM. Insights into the regulation of protein abundance from proteomic and transcriptomic analyses. Nat Rev Genet. 2012;13:227–32. 22411467 10.1038/nrg3185 PMC3654667

[49] Helft J, Bottcher J, Chakravarty P, Zelenay S, Huotari J, Schraml BU, et al. GM-CSF Mouse Bone Marrow Cultures Comprise a Heterogeneous Population of CD11c(+)MHCII(+) Macrophages and Dendritic Cells. Immunity. 2015;42:1197–211. 26084029 10.1016/j.immuni.2015.05.018

[50] Naik SH, Proietto AI, Wilson NS, Dakic A, Schnorrer P, Fuchsberger M, et al. Cutting edge: generation of splenic CD8+ and CD8- dendritic cell equivalents in Fms-like tyrosine kinase 3 ligand bone marrow cultures. J Immunol. 2005;174:6592–7. 15905497 10.4049/jimmunol.174.11.6592

[51] Brasel K, De Smedt T, Smith JL, Maliszewski CR. Generation of murine dendritic cells from flt3-ligand–supplemented bone marrow cultures. Blood. 2000;96:3029–39. 11049981

[52] Morrissey MA, Kern N, Vale RD. CD47 Ligation Repositions the Inhibitory Receptor SIRPA to Suppress Integrin Activation and Phagocytosis. Immunity. 2020;53:290–302.e6. 32768386 10.1016/j.immuni.2020.07.008 PMC7453839

[53] Gong YN, Guy C, Olauson H, Becker JU, Yang M, Fitzgerald P, et al. ESCRT-III Acts Downstream of MLKL to Regulate Necroptotic Cell Death and Its Consequences. Cell. 2017;169:286–300.e16. 28388412 10.1016/j.cell.2017.03.020 PMC5443414

[54] Hanggi K, Ruffell B. Protocol for specific induction of apoptosis or necroptosis in established murine tumors. STAR Protoc. 2025;6:104185. 41205180 10.1016/j.xpro.2025.104185 PMC12766399

[55] Plebanek MP, Xue Y, Nguyen YV, DeVito NC, Wang X, Holtzhausen A, et al. A lactate-SREBP2 signaling axis drives tolerogenic dendritic cell maturation and promotes cancer progression. Sci Immunol. 2024;9:eadi4191. 38728412 10.1126/sciimmunol.adi4191 PMC11926670

[56] Guak H, Al Habyan S, Ma EH, Aldossary H, Al-Masri M, Won SY, et al. Glycolytic metabolism is essential for CCR7 oligomerization and dendritic cell migration. Nat Commun. 2018;9:2463. 29941886 10.1038/s41467-018-04804-6 PMC6018630

[57] Thwe PM, Pelgrom LR, Cooper R, Beauchamp S, Reisz JA, D’Alessandro A, et al. Cell-Intrinsic Glycogen Metabolism Supports Early Glycolytic Reprogramming Required for Dendritic Cell Immune Responses. Cell Metab. 2017;26:558–67 e5. 28877459 10.1016/j.cmet.2017.08.012 PMC5657596

[58] Lei X, Khatri I, de Wit T, de Rink I, Nieuwland M, Kerkhoven R, et al. CD4(+) helper T cells endow cDC1 with cancer-impeding functions in the human tumor micro-environment. Nat Commun. 2023;14:217. 36639382 10.1038/s41467-022-35615-5 PMC9839676

[59] Lei X, de Groot DC, Welters MJP, de Wit T, Schrama E, van Eenennaam H, et al. CD4(+) T cells produce IFN-I to license cDC1s for induction of cytotoxic T-cell activity in human tumors. Cell Mol Immunol. 2024;21:374–92. 38383773 10.1038/s41423-024-01133-1 PMC10978876

[60] Deng L, Liang H, Xu M, Yang X, Burnette B, Arina A, et al. STING-Dependent Cytosolic DNA Sensing Promotes Radiation-Induced Type I Interferon-Dependent Antitumor Immunity in Immunogenic Tumors. Immunity. 2014;41:843–52. 25517616 10.1016/j.immuni.2014.10.019 PMC5155593

[61] Busselaar J, Sijbranda M, Borst J. The importance of type I interferon in orchestrating the cytotoxic T-cell response to cancer. Immunol Lett. 2024;270:106938. 39490629 10.1016/j.imlet.2024.106938

[62] Thorsson V, Gibbs DL, Brown SD, Wolf D, Bortone DS, Ou Yang TH, et al. The Immune Landscape of Cancer. Immunity. 2018;48:812–30.e14. 29628290 10.1016/j.immuni.2018.03.023 PMC5982584

[63] Luca BA, Steen CB, Matusiak M, Azizi A, Varma S, Zhu C, et al. Atlas of clinically distinct cell states and ecosystems across human solid tumors. Cell. 2021;184:5482–96.e28. 34597583 10.1016/j.cell.2021.09.014 PMC8526411

[64] Wellenstein MD, de Visser KE. Cancer-Cell-Intrinsic Mechanisms Shaping the Tumor Immune Landscape. Immunity. 2018;48:399–416. 29562192 10.1016/j.immuni.2018.03.004

[65] Frijlink E, Bosma DMT, Busselaar J, Battaglia TW, Staal MD, Verbrugge I, et al. PD-1 or CTLA-4 blockade promotes CD86-driven Treg responses upon radiotherapy of lymphocyte-depleted cancer in mice. J Clin Invest. 2024;134:e171154. 10.1172/JCI171154 PMC10940086 38349740

[66] Jiang W, Hua R, Wei M, Li C, Qiu Z, Yang X, et al. An optimized method for high-titer lentivirus preparations without ultracentrifugation. Sci Rep. 2015;5:13875. 26348152 10.1038/srep13875 PMC4562269

[67] Vremec D, Hansen J, Strasser A, Acha-Orbea H, Zhan Y, O’Keeffe M, et al. Maintaining dendritic cell viability in culture. Mol Immunol. 2015;63:264–7. 25081090 10.1016/j.molimm.2014.07.011

