# Supplementary material for: Phagocytosis of necroptotic cells optimizes type 1 conventional dendritic cells for induction of a cytotoxic T-cell response
Source: Cell Death Differ. 2026 Feb 28;33(9):1768–82. doi: 10.1038/s41418-026-01689-7 (PMC13545219; doi:10.1038/s41418-026-01689-7)
Supplement: Supplementary file 1 [file 41418_2026_1689_MOESM1_ESM.pdf]

SUPPLEMENTARY INFORMATION

Phagocytosis of necroptotic cells optimizes type 1 conventional dendritic cells for induction of a cytotoxic T-cell response

Mo D. Staal[1,2], Zhixian Wang[1], Douwe M. T. Bosma[1,2], Julia Busselaar[1,2], Ellen Schrama[1,2], Evert de Vries[1,2], Yanling Xiao[1,2] and Jannie Borst[1,2]

[1]Department of Immunology and [2]Oncode Institute, Leiden University Medical Center, Leiden, The Netherlands

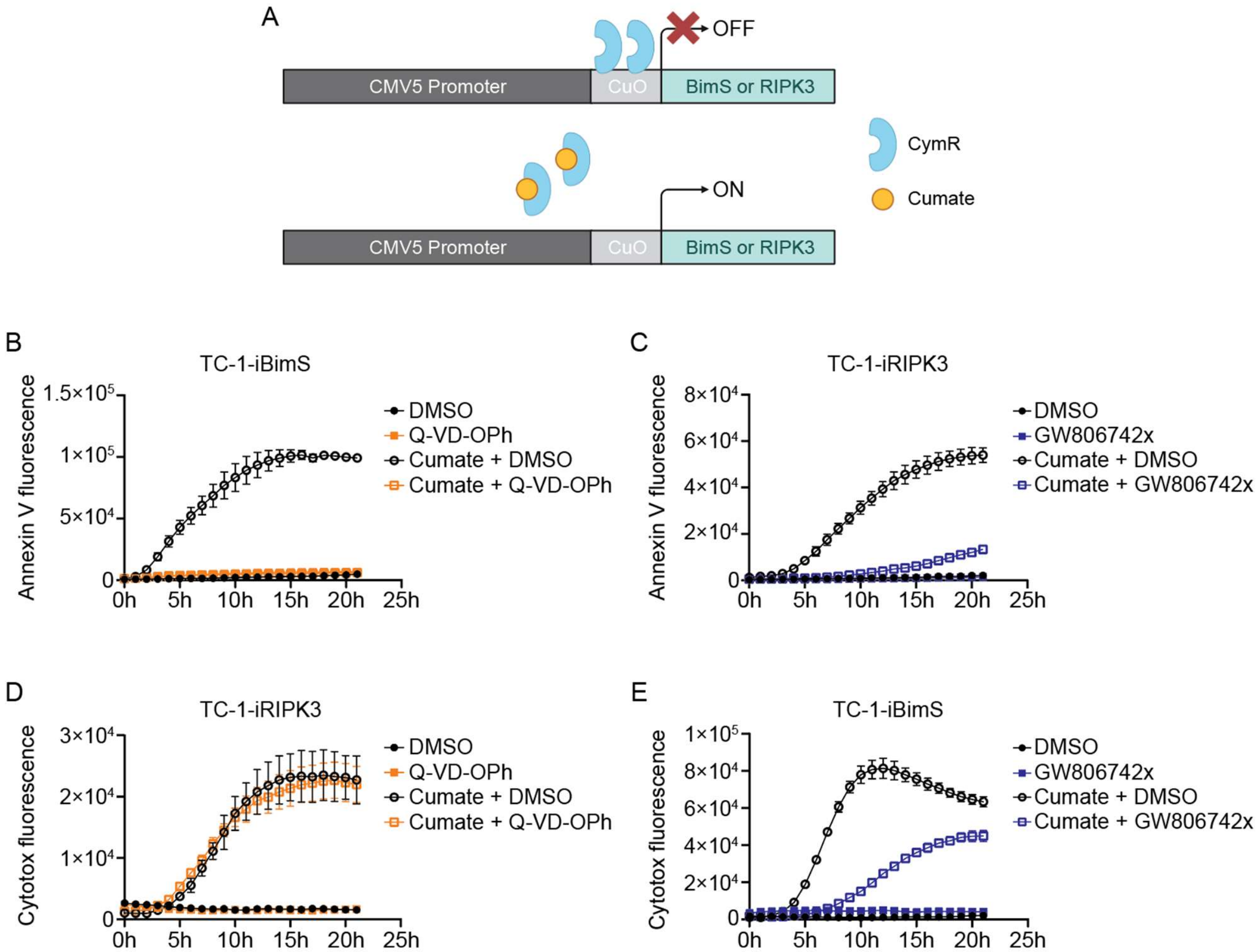

Supplementary Figure 1 (belonging to Figure 1)**.**

Validation of apoptosis and necroptosis induction in engineered TC-1 tumor cells.

(A) Schematic overview of the cumate gene switch. The repressor protein CymR, introduced by lentiviral transduction, is constitutively expressed and binds to the cumate operon (CuO) to prevent transcription of the target gene BimS or RIPK3 downstream of the CMV5 promoter. The presence of cumate disrupts the binding of CymR to the CuO and allows for efficient transcription of BimS or RIPK3 leading to apoptosis or necroptosis, respectively. **(B-E)** Real-time Incucyte analysis of Annexin V dye fluorescence in TC-1-iBimS cells (B) or TC-1-iRIPK3 cells (C), or Cytotox Dye incorporation in TC-1-iRIPK3 cells (D) or TC-1-iBimS cells (E) during indicated treatments with vehicle (DMSO), or pan-caspase inhibitor Q-VD-OPh, or MLKL inhibitor GW806742x, in presence or absence of inducer cumate. Means ± SD of technical triplicates are shown, representative of two independent experiments.

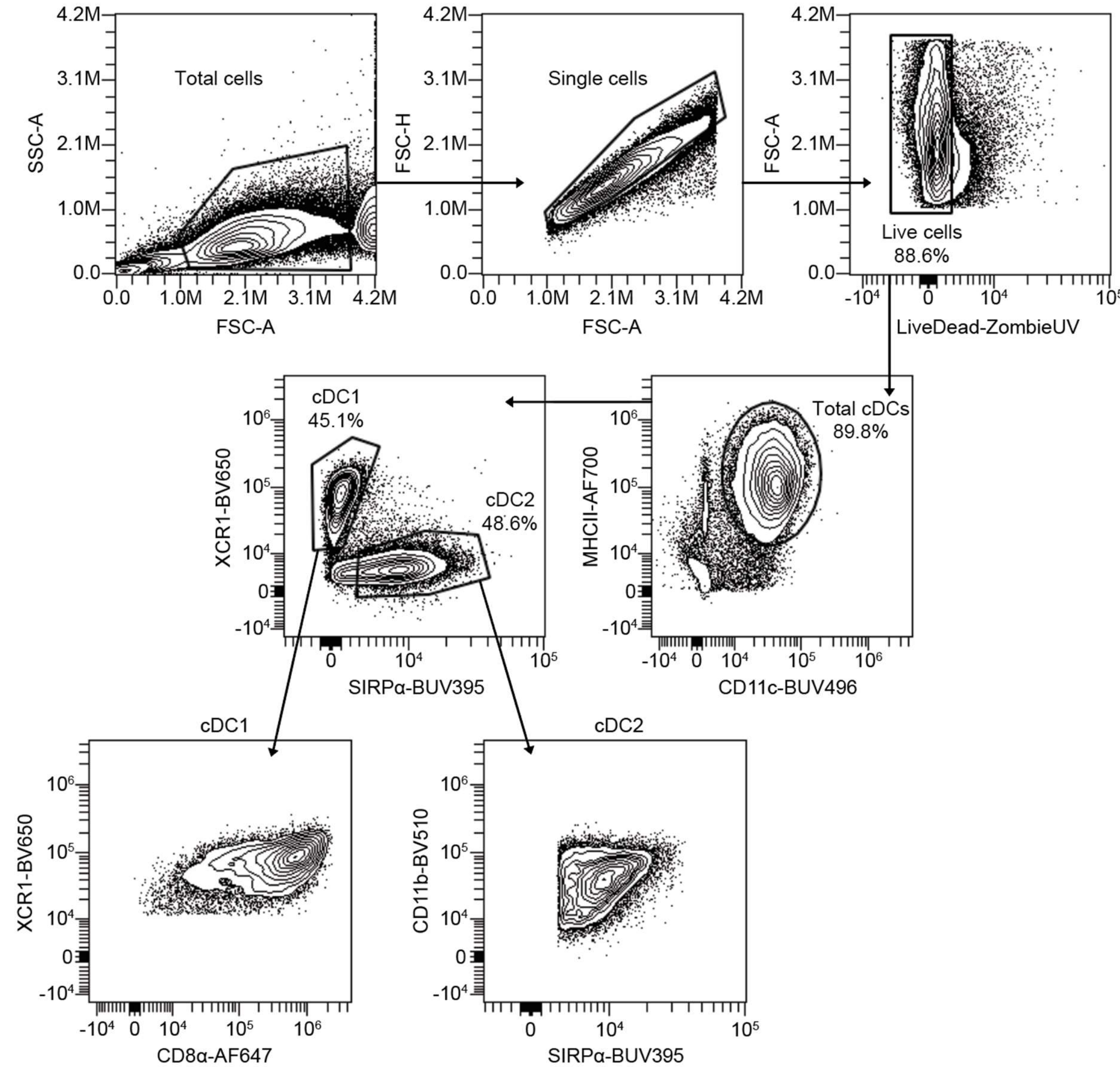

Supplementary Figure 2 (belonging to all cDC1 and cDC2 analyses, except Fig. S10, S11)**.**

Flow cytometry gating strategy to identify cDC1s and cDC2s.

Among purified CD11c$^+$ cells from mouse spleen, cDC1 and cDC2 populations were identified by the following gating strategy: 1) debris was excluded based on side scatter area (SSC-A) and forward scatter (FSC)-A; 2) cell doublets were excluded based on FSC height (FSC-H) and FSC-A; 3) dead cells were excluded based on ZombieUV staining; 4) the total splenic cDC population was gated based on MHC-II$^+$CD11c$^+$ phenotype; 5) cDC1s were identified by XCR1 and CD8α co-expression and lack of SIRPα, and cDC2s were identified by SIRPα and CD11b co-expression and lack of XCR1. Representative percentages of gated cell populations are shown.

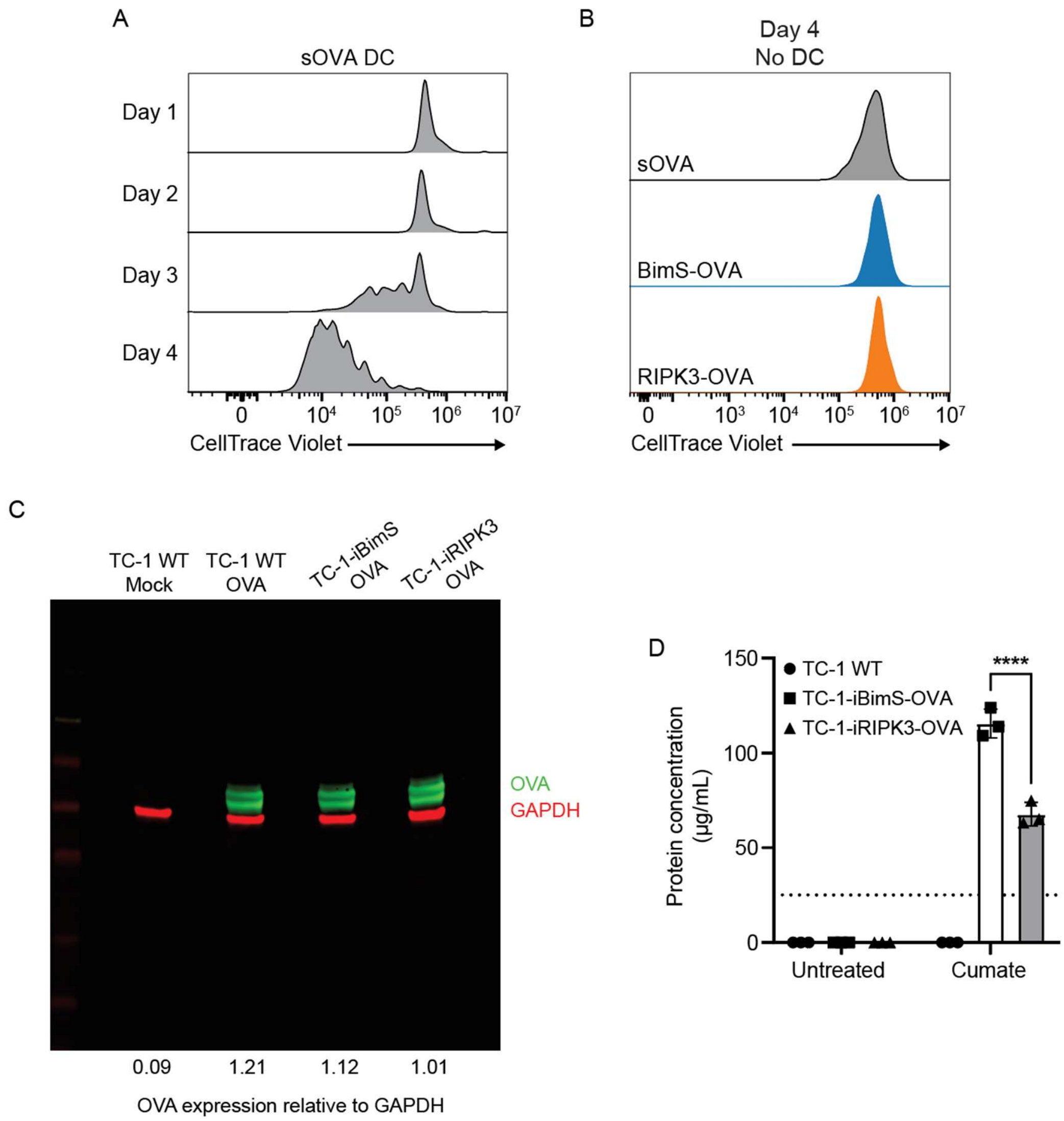

Supplementary Figure 3 (belonging to Figure 2)**.**

Validation of the cDC-OT-I co-culture platform and OVA-expressing TC-1 cells.

(A, B) Flow cytometry histograms showing CellTrace Violet (CTV) dilution in ovalbumin (OVA)-specific OT-I T cells after co-culture for the indicated days with cDCs loaded with full-length soluble (s)OVA (A), or and after co-culture for 4 days with sOVA, TC-1-iBimS-OVA or TC-1-iRIPK3-OVA cell debris in absence of cDCs (B). Data are representative of three independent experiments. **(C)** Western Blot showing full-length OVA and GAPDH expression in indicated cell lines after lentiviral transduction with empty vector (mock) or OVA-encoding vector. Quantification of OVA protein level relative to GAPDH protein level in each lane is depicted underneath the blot. Western Blot shown is uncropped. **(D)** Bar graph showing protein concentration of medium from indicated cell lines cultured for 24 h in serum-free Opti-MEM with or without cumate, as determined by BCA protein quantification assay. Means ± SD of three independent experiments are shown. ****$P < 0.0001$; one-way ANOVA with Tukey's multiple comparisons test.

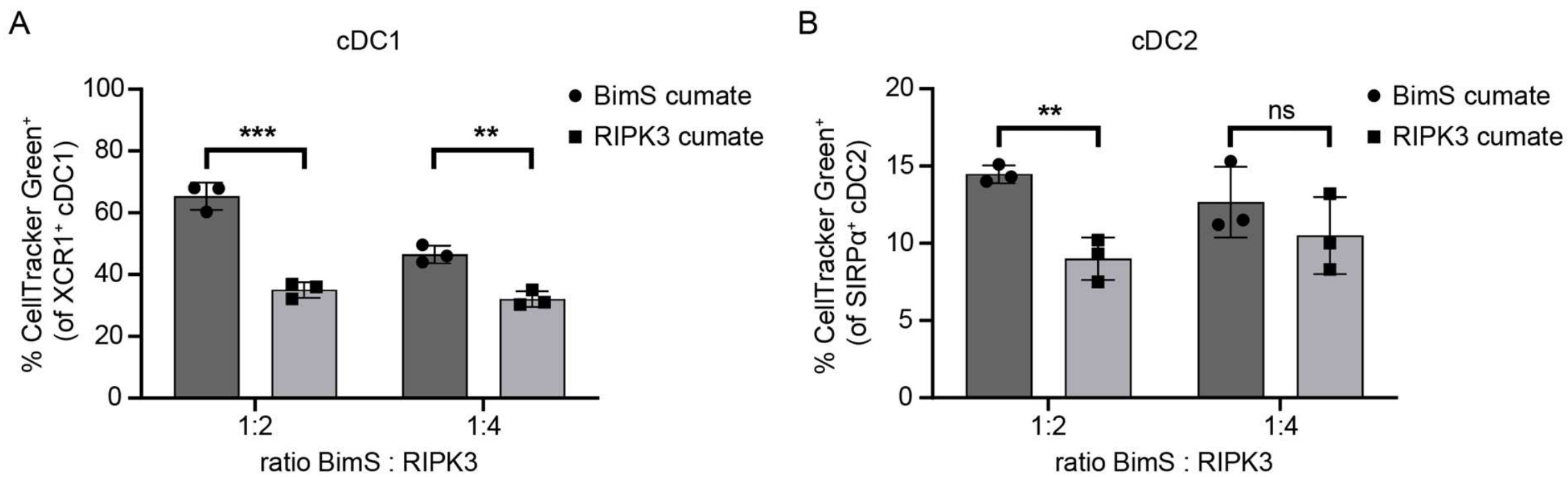

Supplementary Figure 4 (belonging to Figure 5)**.**

Phagocytosis of apoptotic and necroptotic TC-1 tumor cells by cDC1s and cDC2s.

cDCs were presented with debris from indicated CellTracker Green (CTG)+ apoptotic and necroptotic TC-1 tumor cells after cumate induction, in 1:2 or 1:4 ratio. Phagocytosis was determined by flow cytometric detection of CTG in cDC subsets. Bar graphs show the frequency of CTG+ cDC1s **(A)** and cDC2s **(B)** after 6 h of co-culture. Means ± SD of three independent experiments are shown. ns, not significant; **P < 0.01; ***P < 0.001; unpaired Student's t-test.

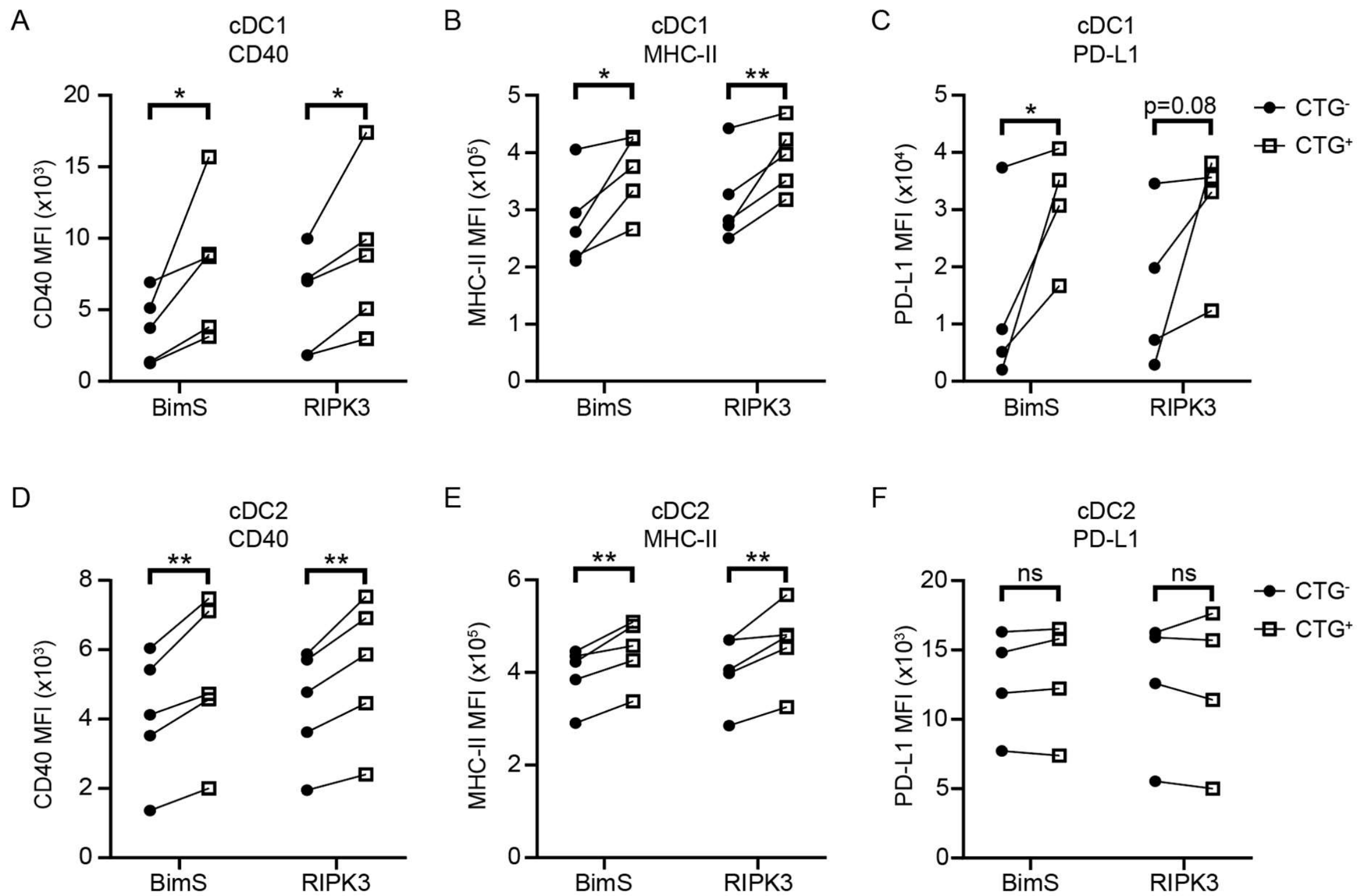

Supplementary Figure 5 (belonging to Figure 6)**.**

Changes in cell surface protein profile of cDC1s and cDC2s upon phagocytosis of apoptotic or necroptotic TC-1 tumor cells.

cDCs were presented with debris from indicated CTG^+ apoptotic or necroptotic TC-1 tumor cells after cumate induction as outlined in Figure 5. Graphs show the median fluorescence intensity (MFI) of CD40, MHC-II, and PD-L1 between the cDC1s **(A-C)** and cDC2s **(D-F)** in the same sample that had not (CTG^-) or had (CTG^+) phagocytosed CTG^+ TC-1 cells. Each individual value within a graph belongs to an independent experiment. ns, not significant; *$P < 0.05$; **$P < 0.01$; one-tailed paired Student's t-test.

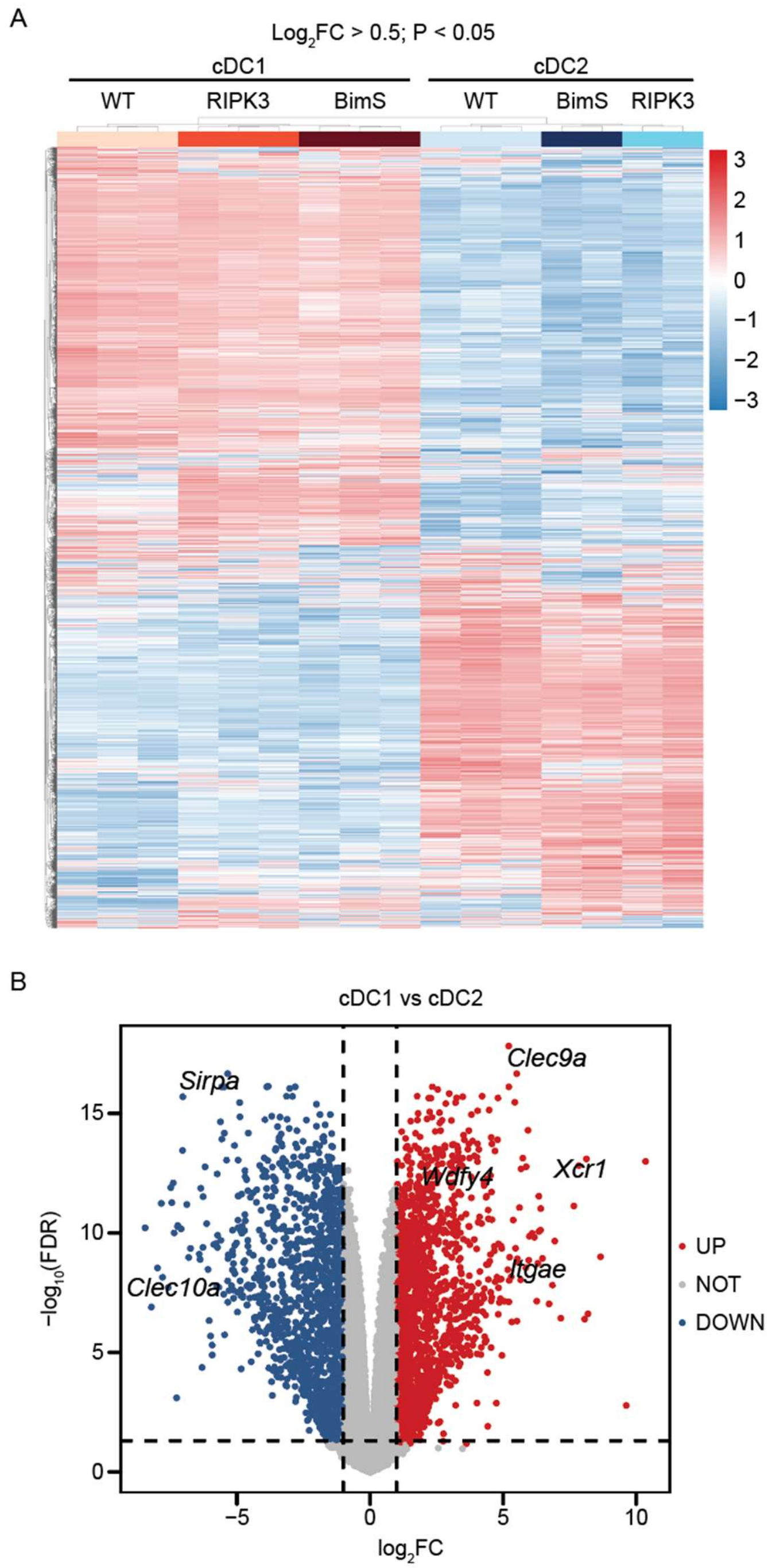

Supplementary Figure 6 (belonging to Figure 7)**.**

Confirmation of cDC1 and cDC2 identity in bulk RNAseq data.

MACS-purified CD11c+ cells were cultured with medium from wild-type (WT) TC-1 cells or debris from apoptotic (BimS) or necroptotic (RIPK3) TC-1 cells for 6 h. cDC1 and cDC2 were subsequently flow cytometrically purified, based on the gating strategy in Fig. S2, and analyzed by bulk RNAseq as outlined for Figure 7. (**A**) Heatmap after hierarchical clustering of identified transcripts. The gene list to generate the heatmap was based on a P value < 0.05 and a $Log_2FC > 0.5$. (**B**) Volcano plot comparing transcripts from cDC1s and cDC2s.

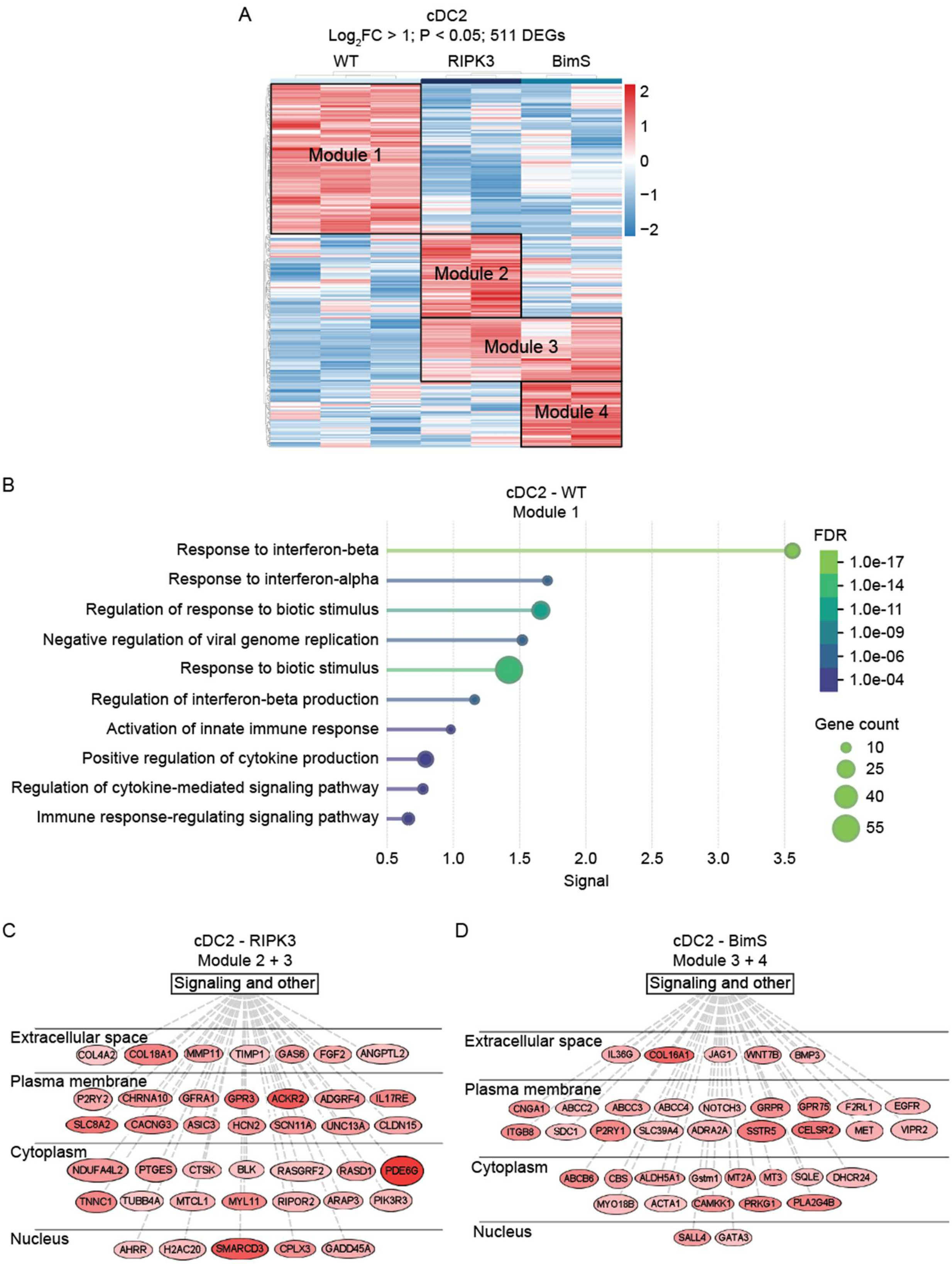
A
cDC2
Log2FC > 1; P < 0.05; 511 DEGs
WT
RIPK3
BimS
Module 1
Module 2
Module 3
Module 4
2
1
0
−1
−2
B
cDC2 - WT
Module 1
Response to interferon-beta
Response to interferon-alpha
Regulation of response to biotic stimulus
Negative regulation of viral genome replication
Response to biotic stimulus
Regulation of interferon-beta production
Activation of innate immune response
Positive regulation of cytokine production
Regulation of cytokine-mediated signaling pathway
Immune response-regulating signaling pathway
0.5
1.0
1.5
2.0
2.5
3.0
3.5
Signal
FDR
1.0e-17
1.0e-14
1.0e-11
1.0e-09
1.0e-06
1.0e-04
Gene count
10
25
40
55
C
cDC2 - RIPK3
Module 2 + 3
Signaling and other
Extracellular space
COL4A2
COL18A1
MMP11
TIMP1
GAS6
FGF2
ANGPTL2
Plasma membrane
P2RY2
CHRNA10
GFRA1
GPR3
ACKR2
ADGRF4
IL17RE
SLC8A2
CACNG3
ASIC3
HCN2
SCN11A
UNC13A
CLDN15
Cytoplasm
NDUFA4L2
PTGES
CTSK
BLK
RASGRF2
RASD1
PDE6G
TNNC1
TUBB4A
MTCL1
MYL11
RIPOR2
ARAP3
PIK3R3
Nucleus
AHRR
H2AC20
SMARCD3
CPLX3
GADD45A
D
cDC2 - BimS
Module 3 + 4
Signaling and other
Extracellular space
IL36G
COL16A1
JAG1
WNT7B
BMP3
Plasma membrane
CNGA1
ABCC2
ABCC3
ABCC4
NOTCH3
GRPR
GPR75
F2RL1
EGFR
ITGB8
SDC1
P2RY1
SLC39A4
ADRA2A
SSTR5
CELSR2
MET
VIPR2
Cytoplasm
ABCB6
CBS
ALDH5A1
Gstm1
MT2A
MT3
SQLE
DHCR24
MYO18B
ACTA1
CAMKK1
PRKG1
PLA2G4B
Nucleus
SALL4
GATA3

Supplementary Figure 7 (belonging to Figure 7)**.**

Transcriptomic response of cDC2s to apoptotic or necroptotic TC-1 tumor cells.

MACS-purified CD11c⁺ cells were cultured with medium from wild-type (WT) TC-1 cells or debris from apoptotic (BimS) or necroptotic (RIPK3) TC-1 cells for 6 h. cDC1 and cDC2 were subsequently flow cytometrically purified, based on the gating strategy in Fig. S2, and analyzed by bulk RNAseq as outlined for Figure 7. Depicted are analyses of RNAseq data from cDC2s. **(A)** Hierarchical clustering in heatmap of differentially expressed genes (DEGs). The gene list to generate the heatmap was based on a P value < 0.05 and a $Log_2FC > 1$. Module 1: 209 genes; module 2: 119 genes; module 3: 57 genes; module 4: 123 genes. **(B)** STRING Gene Ontology analysis of genes upregulated in WT-enriched module 1. Similarity filter cutoff value >= 0.4 was used. **(C, D)** IPA of genes upregulated in RIPK3-enriched modules 2 and 3 (C) or genes upregulated in BimS-enriched modules 3 and 4 (D). Genes are clustered based on subcellular localization. Red color gradient is based on relative expression level.

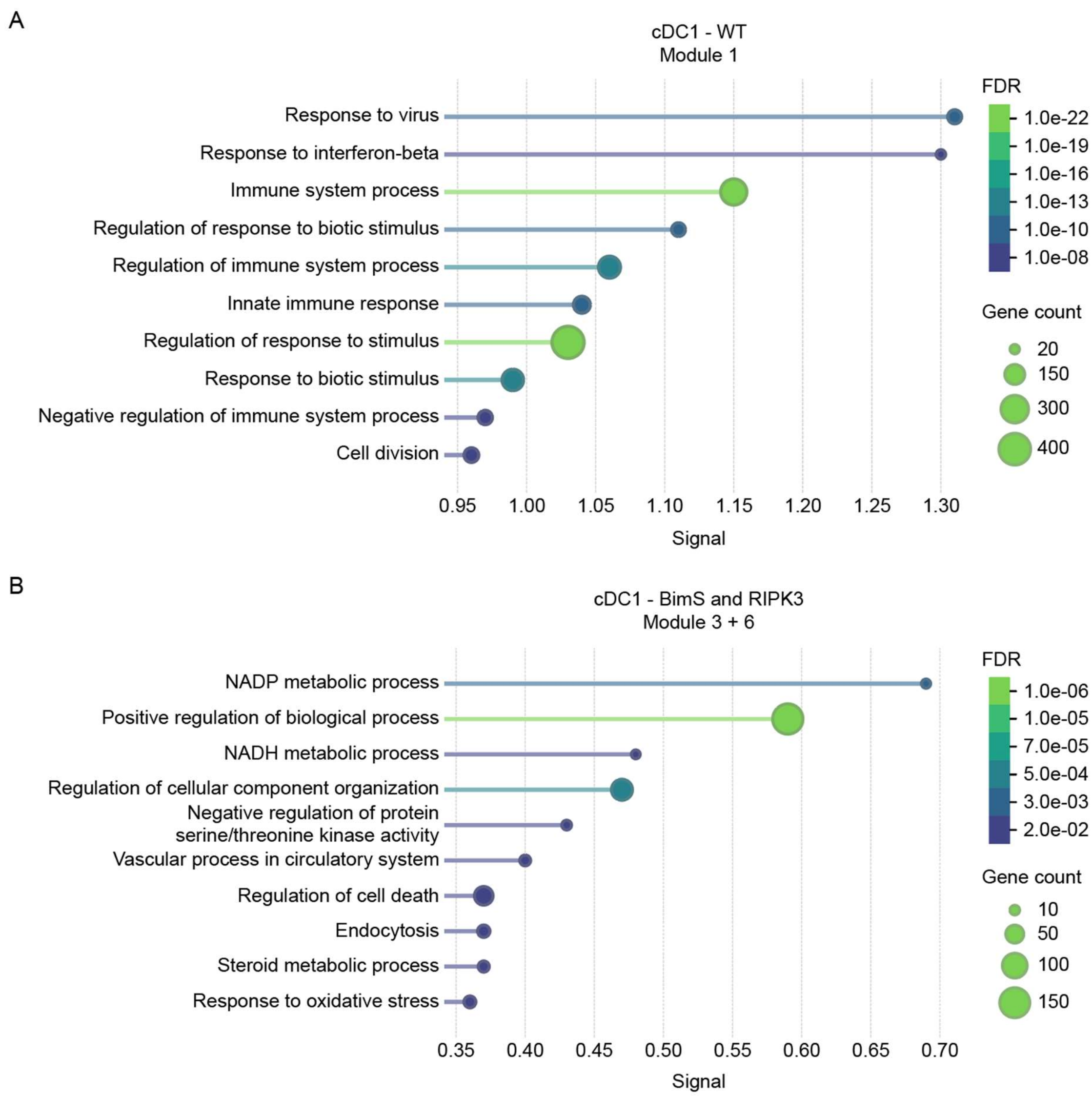

Supplementary Figure 8 (belonging to Figure 7)**.**

Transcriptomic response of cDC1s to apoptotic or necroptotic TC-1 tumor cells.

MACS-purified CD11c$^+$ cells were cultured with medium from wild-type (WT) TC-1 cells or debris from apoptotic (BimS) or necroptotic (RIPK3) TC-1 cells for 6 h. cDC1 and cDC2 were subsequently flow cytometrically purified, based on the gating strategy in Fig. S2, and analyzed by bulk RNAseq as outlined for Figure 7. Depicted are analyses of RNAseq data from cDC1s. **(A, B)** STRING Gene Ontology analysis of upregulated genes in WT-enriched module 1 (A) or upregulated genes of shared modules 3 and 6 (B). Similarity filter cutoff value >= 0.4 was used.

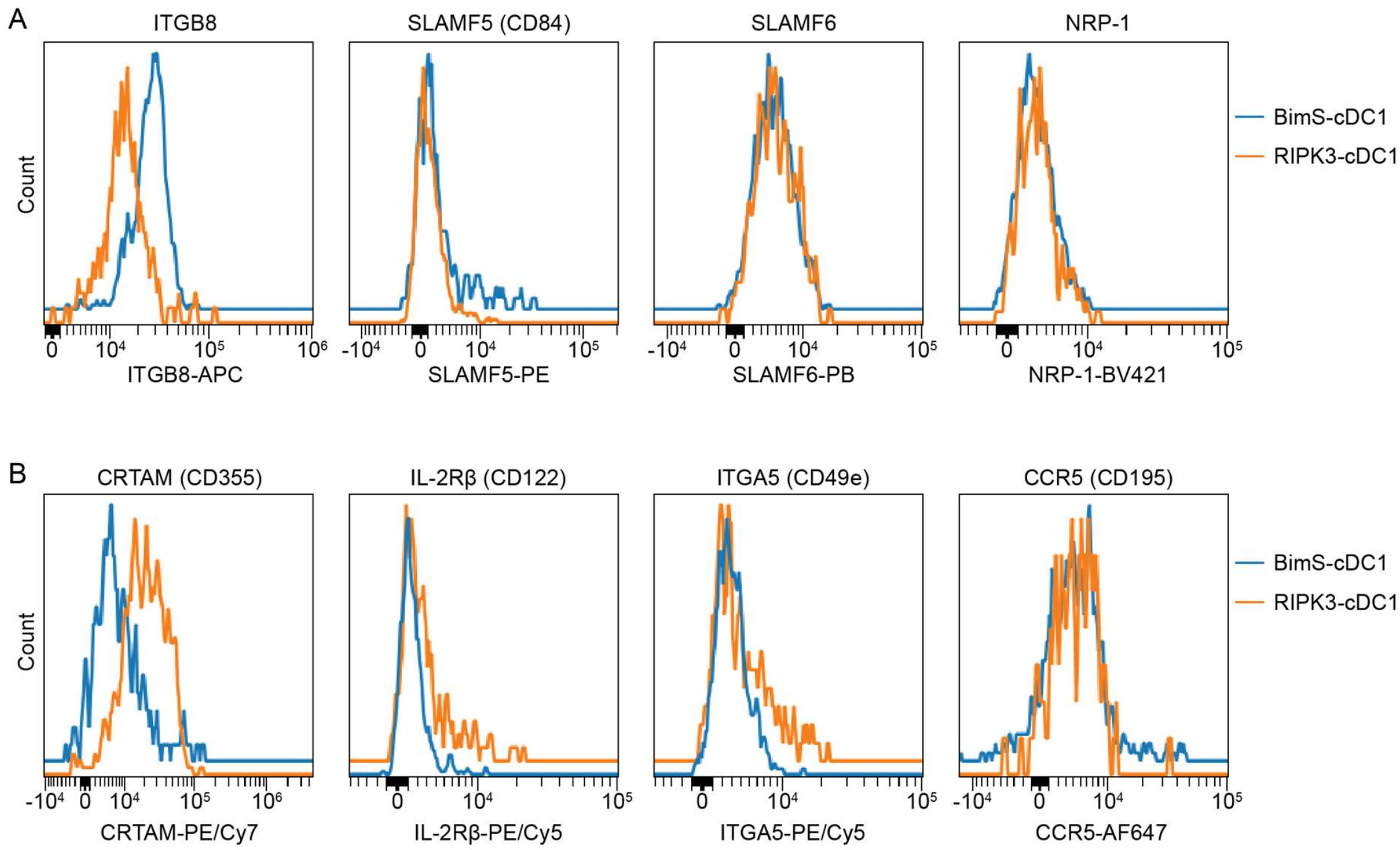

Supplementary Figure 9.

Differential cell surface protein levels in cDC1s after phagocytosing apoptotic or necroptotic cells.

MACS-purified CD11c⁺ cells were cultured with debris from apoptotic (BimS) or necroptotic (RIPK3) TC-1 cells for 6 h as described in Fig. 5A. cDC1s were identified by spectral flow cytometry based on the gating strategy depicted in Fig. S2. **(A, B)** Flow cytometry histograms showing cell surface protein levels of molecules that had in our bulk RNAseq dataset a higher mRNA level in cDC1s that had engulfed apoptotic TC-1 cells (A) or in cDC1s that had engulfed necroptotic TC-1 cells (B).

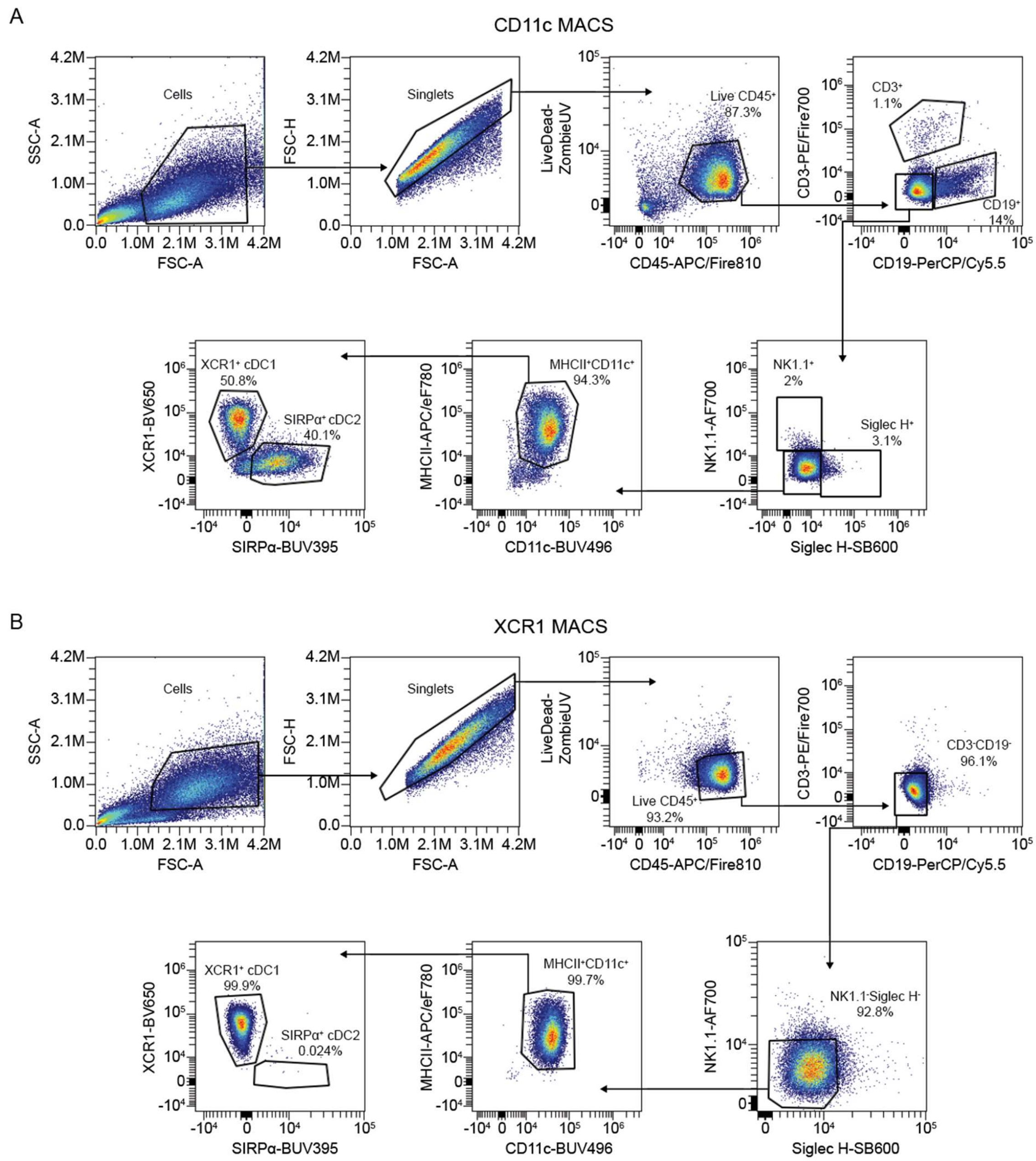

Supplementary Figure 10 (belonging to Supplementary Figure 11).

Flow cytometry gating strategy to identify CD11c$^+$ and XCR1$^+$ cell populations.

(A, B) Among MACS-purified CD11c$^+$ (A) or MACS-purified XCR1$^+$ (B) cells from mouse spleen, cDC1 and cDC2 populations were identified by the following gating strategy: 1) debris was excluded based on side scatter area (SSC-A) and forward scatter (FSC)-A; 2) cell doublets were excluded based on FSC height (FSC-H) and FSC-A; 3) live immune cells were defined by negative ZombieUV and positive CD45 staining; 4) CD3$^+$ and CD19$^+$ cells were excluded; 5) NK1.1$^+$ and Siglec H$^+$ populations were excluded; 6) the total splenic cDC population was gated based on MHC-II$^+$CD11c$^+$ phenotype; 5) cDC1s were identified by XCR1 expression and lack of SIRPα, and cDC2s were identified by SIRPα expression and lack of XCR1. Representative percentages of gated cell populations are shown.

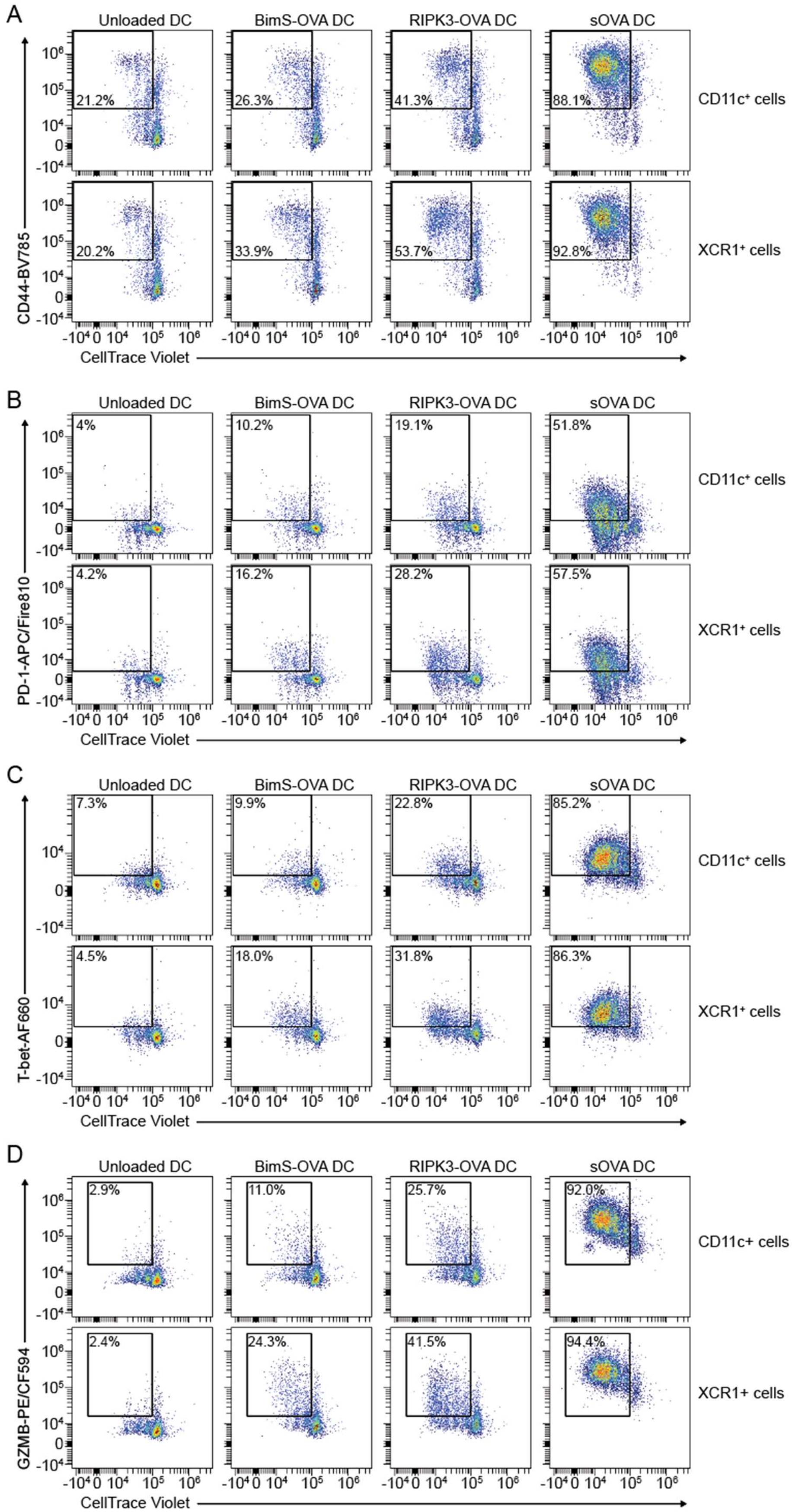
A
Unloaded DC
BimS-OVA DC
RIPK3-OVA DC
sOVA DC
21.2%
26.3%
41.3%
88.1%
CD11c⁺ cells
20.2%
33.9%
53.7%
92.8%
XCR1⁺ cells
CD44-BV785
CellTrace Violet
B
4%
10.2%
19.1%
51.8%
4.2%
16.2%
28.2%
57.5%
PD-1-APC/Fire810
C
7.3%
9.9%
22.8%
85.2%
4.5%
18.0%
31.8%
86.3%
T-bet-AF660
D
2.9%
11.0%
25.7%
92.0%
CD11c+ cells
2.4%
24.3%
41.5%
94.4%
XCR1+ cells
GZMB-PE/CF594

Supplementary Figure 11.

Comparison of the cDC-OT-I co-culture platform with CD11c⁺ or XCR1⁺ cells.

The cDC-OT-I co-culture platform was used as described in Fig. 2A with either MACS-isolated splenic CD11c$^+$ cells or MACS-isolated splenic XCR1$^+$ cells, and the phenotype of OT-I cells was read out at day 3 by flow cytometry. **(A-D)** Representative flow cytometry scatterplots depicting the CTV$^-$CD44$^+$ (A), CTV$^-$PD-1$^+$ (B), CTV$^-$T-bet$^+$ (C), or CTV$^-$GZMB$^+$ (D) OT-I cells. Representative percentages of gated cell populations are shown, and data are representative of two independent experiments.

Movie Titles

Movie 1. Cytotox Dye incorporation by dying TC-1-iBimS cells

Movie 2. TC-1-iBimS cell death inhibition by pan-caspase inhibitor Q-VD-OPh

Movie 3. Cytotox Dye incorporation by dying TC-1-iRIPK3 cells

Movie 4. TC-1-iRIPK3 cell death inhibition by MLKL inhibitor GW806742x

Movie 5. MLKL inhibitor GW806742x does not block cell death of TC-1-iBimS cells
